# Mutational signatures reveal ternary relationships between homologous recombination repair, APOBEC, and mismatch repair in gynecological cancers

**DOI:** 10.1186/s12967-022-03259-0

**Published:** 2022-02-02

**Authors:** Amir Farmanbar, Sanaz Firouzi, Robert Kneller, Hossein Khiabanian

**Affiliations:** 1 Center for Systems and Computational Biology, Rutgers Cancer Institute of New Jersey, Rutgers University, New Brunswick, NJ 08903 USA; 2 Institute for Cancer Genetics, Columbia University, New York, NY 10032 USA; 3 Research Center for Advanced Science and Technology, University of Tokyo, Minato-ku, Tokyo, 153-8904 Japan; 4 Department of Pathology and Laboratory Medicine, Rutgers Robert Wood Johnson Medical School, Rutgers University, New Brunswick, NJ 08903 USA

**Keywords:** Gynecological cancers, Uterine corpus endometrial carcinoma, Ovarian cancer, Cervical cancer, Mutational Signature, DNA damage and repair, MMR, NHEJ, HR, APOBEC, POLE, Tumor mutational burden

## Abstract

**Background:**

Revealing the impacts of endogenous and exogenous mutagenesis processes is essential for understanding the etiology of somatic genomic alterations and designing precise prognostication and treatment strategies for cancer. DNA repair deficiency is one of the main sources of endogenous mutagenesis and is increasingly recognized as a target for cancer therapeutics. The role and prevalence of mechanisms that underly different forms of DNA repair deficiencies and their interactions remain to be elucidated in gynecological malignancies.

**Methods:**

We analyzed 1231 exomes and 268 whole-genomes from three major gynecological malignancies including uterine corpus endometrial carcinoma (UCEC) as well as ovarian and cervical cancers. We also analyzed data from 134 related cell lines. We extracted and compared de novo and refitted mutational signature profiles using complementary and confirmatory approaches and performed interaction analysis to detect co-occurring and mutually exclusive signatures.

**Results:**

We found an inverse relationship between homologous recombination deficiency (HRd) and mismatch repair deficiency (MMRd). Moreover, APOBEC co-occurred with HRd but was mutually exclusive with MMRd. UCEC tumors were dominated by MMRd, yet a subset of them manifested the HRd and APOBEC signatures. Conversely, ovarian tumors were dominated by HRd, while a subset represented MMRd and APOBEC. In contrast to both, cervical tumors were dominated by APOBEC with a small subsets showing the POLE, HRd, and MMRd signatures. Although the type, prevalence, and heterogeneity of mutational signatures varied across the tumor types, the patterns of co-occurrence and exclusivity were consistently observed in all. Notably, mutational signatures in gynecological tumor cell lines reflected those detected in primary tumors.

**Conclusions:**

Taken together, these analyses indicate that application of mutation signature analysis not only advances our understanding of mutational processes and their interactions, but also it has the potential to stratify patients that could benefit from treatments available for tumors harboring distinct mutational signatures and to improve clinical decision-making for gynecological malignancies.

**Supplementary Information:**

The online version contains supplementary material available at 10.1186/s12967-022-03259-0.

## Background

DNA-damaging agents and deficiency in DNA repair system result in an increased rate of somatic mutation, and therefore, are causative factors for carcinogenesis [1, 2]. Tumor mutational burden (TMB) and mutational signatures are a reflection of the processes that give rise to mutation accumulation during tumor development and progression [3]. While TMB simply approximates the number of somatic alterations, mutational signatures infer mutational fingerprints and can elaborate multiple cancer processes in effect [4], to provide novel insights into tumor etiology [5].

Uterine, ovarian and cervical cancers are the most common types of gynecological tumors with heterogeneous underlying etiology and genomic structure [6–8]. While there has been extensive investigation of mismatch repair deficiency (MMRd) in uterine corpus endometrial carcinoma (UCEC), MMRd in ovarian and cervical cancers is relatively under-investigated [9, 10]. Conversely, homologous recombination deficiency (HRd) has been mainly investigated in ovarian cancer and it remains relatively underexplored in other gynecological tumors [11, 12]. In this context, the role and prevalence of Apolipoprotein B mRNA editing enzyme, catalytic polypeptide-like (APOBEC), which has been extensively studied in lung and breast cancers has remained unexplored in gynecological malignancies [13, 14].

MMRd leads to increased mutation load, which in turn may impact anti-tumor immune responses and treatment effectiveness [15, 16]. Currently, there are a number of mutational signatures described in primary cancers associated with MMRd [4, 17]. Moreover, several studies have demonstrated the efficacy of immunotherapy for treating tumors with microsatellite instability (MSI) phenotype [18, 19]. In turn, defective MMR accompanied by MSI has become an important biomarker for therapeutic selection in diverse tumor types, including colorectal, gastric, endometrial cancers [20, 21]; therefore, evaluation of MMRd in gynecological cancers via mutational signature analysis may help stratify patients suitable for immunotherapy.

Similarly, HRd has become a demonstrated biomarker for identifying patients who may benefit from treatments that target this defect, including poly (ADP-ribose) polymerase(PARP) inhibitors and platinum therapy [22, 23]. HR is responsible for the repair of double-stranded DNA breaks [24], which may be caused by endogenous stresses arising from cellular metabolism, such as replication stress and reactive oxygen species (ROS), as well as exogenous factors, such as ionizing radiation and chemotherapy agents [25]. About half of ovarian tumors present defective DNA repair related to the HR pathway [26]; however, the effects and interactions of HRd on other DNA repair mechanisms such as MMR has remained unknown.

Dysregulation of physiological mutagenesis processes in cancer cells may also contribute to accumulation of mutations. For example, aberrant activity of APOBEC enzymes, which are involved in protection against viral infections, has been shown as a major source of mutations in multiple human cancer types [27, 28], including bladder, cervix, lung, head and neck, and breast [4, 17, 29–31]. Currently, ABOBEC3A and APOBEC3B are the main family members implicated in cancer [31, 32], and SBS2 and SBS13 are mutational signatures associated with APOBEC-mediated mutagenesis [4, 17]. In particular, APOBEC3 enzymes are being studied as a therapeutic target for chemicals or molecular therapies that harness associated mutational processes [28]. Exploring the prevalence of APOBEC and its interactions with DNA repair systems may help clarify mechanisms of mutagenesis in gynecological cancers and suggest possible therapies.

In this study, we analyzed whole genome sequencing (WGS) and whole exome sequencing (WES) data to infer active mutational signatures in gynecological tumors as well as their related cell lines. We estimated the contributions of each mutational signature to each tumor, compared the numbers of mutations attributable to the signatures, and analyzed interactions between different mutational signatures associated with DNA repair and damage pathways. Our results suggest individualized mutational signature-based therapeutic possibilities for gynecological cancers as well as insights for understanding interactions between different DNA repair/damage mechanisms.

## Methods

### Mutation data source and cleaning

We accessed, cleaned, and reformatted Simple Somatic Mutation (SSM) files for WGS and WES of primary tumors from the data portal of the International Cancer Genome Consortium (ICGC, https://dcc.icgc.org/projects) and utilized them for subsequent analyses. WGS data included UCEC (n = 91), ovarian (n = 157) and cervical (n = 20) tumors. WES data included UCEC (n = 531), ovarian (n = 411) from ICGC and cervical (n = 289) tumors from the Cancer Genome Atlas (TCGA). In addition, 134 related gynecological cancer cell lines including UCEC related (n = 40), ovarian related (n = 74) and cervical related (n = 21) were accessed via Cancer Cell Line Encyclopedia (CCLE) [33].

### Mutational signatures analysis

Mutational signatures of WES and WGS of tumor samples were extracted using an NMF-based de novo mutational signatures analysis [34] decomposed by COSMIC, V3.1 which includes 96 Single-Base Substitutions (SBSs), 78 Double-Base Substitutions (DBSs) and 83 small Insertions/Deletions (IDs) signatures (http://cancer.sanger.ac.uk/cosmic/signatures).

The cosine similarity [4, 17], which is the cosine of the angle between two vectors in space, is the standard measure for comparing two spectra. For decomposing the identified NMF-based signatures to COSMIC signatures, cosine similarity was used.

Confirmatory mutational signature analysis was performed using the computational tool SigMA, using hg19 as the reference human genome. SigMA, which uses a likelihood-based approach, has been used to accurately detect the mutational signature associated with HR deficiency (SBS3) from WGS, WES and targeted gene panels, even from low mutation counts. SigMA was run in tuned MVA models [do_mva = T and do_assign = T] for UCEC and ovarian cancer, and in exploratory mode [do_mva = F and do_assign = F] for cervical cancer. For all tumors, run () function was set as check_msi = T and add_sig3 = T. A detailed description of the algorithm and its performance is provided in [35].

TMB was measured as number of all somatic mutations per Megabase (Mb) of each tumor genome, visualized by [34]. Following extraction of mutational signatures for each sample, the first and second dominant signatures were visualized based on their contributions over the exomes or the whole genomes of analyzed tumors. Hierarchical clustering was performed based on the relative contribution of signatures in each tumor. These data were displayed in the clustered heatmaps splitted-up and annotated by their dominant signatures. The contribution values of MMRd, HRd, and APOBEC signatures were used as inputs to draw the ternary plots. All visualizations were performed using the “car”, “ComplexHeatmap”, “stats”,“circlize”, “ggplot”, and “ternary” packages in R [36–38].

### Survival analysis

Overall survival (OS) data were downloaded from ICGC Data Portal(https://dcc.icgc.org) and used for survival analysis utilizing the “Survminer” and “survival” packages in R [39]. *P* values representing the significance were determined from log-rank.

### Statistical analyses

*P* values were calculated by Hypergeometric test for assessing significance of co-occurrences and exclusivity of signatures; and by Fisher exact test for assessing significance of the dominant signature in signature groups. All analyses were conducted in the R statistical environment (R version 4.0.0 http://www.r-project.org/). All reported *P* values were two tailed; ≤ 0.05 was considered significant.

## Results

We performed mutational signature analyses using two independent and complementary approaches. First, we used a non-Negative Matrix Factorization (NMF)-based de novo mutational signature detection method, decomposed by the Catalogue of Somatic Mutations in Cancer (COSMIC, V3.1), and analyzed SBSs, IDs, and DBSs. As a second approach, and to further confirm the results, we used a complimentary, multivariate analysis for extracting SBS mutational signatures. We extracted and compared mutational signatures from WGS data of UCEC (n = 91), ovarian (n = 157), and cervical (n = 20) tumors as well as WES data of UCEC (n = 531), ovarian (n = 411), and cervical (n = 289) tumors. In addition, we analyzed 134 related gynecological cancer cell lines. The results of (NMF)-based de novo mutational signature detection method and multivariate analysis are included in Additional file 1: Table S1 A–X.

### Mutational signatures of UCEC genomes and exomes reveal mutual exclusivity of MMRd and HRd as well as co-occurrence of APOBEC with HRd

In total, we analyzed 455,621 SBSs, 2,940 IDs, and 1,561 DBSs from 91 UCEC whole genomes to detect NMF-based de novo mutational signatures. MMRd signatures, including SBS15 and SBS44, were present in 23% of tumors, with an average contribution of 0.43 (range: 0.2–0.81). MMRd was the first or second dominant signature when present. HRd (SBS3) was present in 14% of tumors with an average contribution of 0.49 (range: 0.36–0.73) as the first or second dominant signature.

**Fig. 1:**
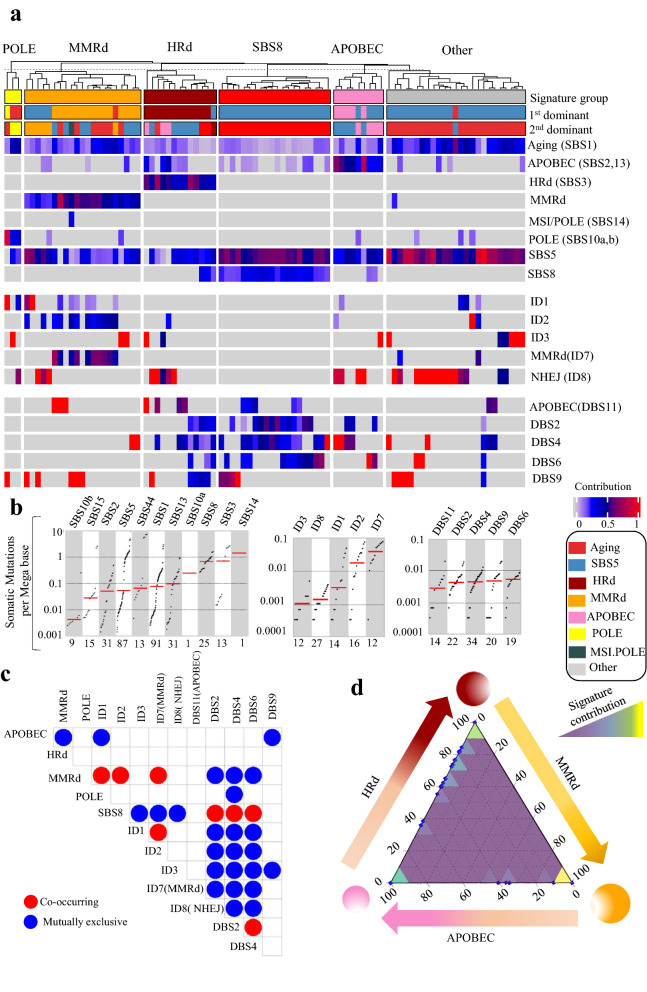
Mutational signature analysis of UCEC WGS tumors. **a** NMF-based de novo Mutational signatures of UCEC tumors visualized by a heatmap divided based on distinct signature status. The first and second dominant signatures are annotated in the top. The dominant signatures were significantly associated with their corresponding signature group (HRd: *P* 4.47E-12, MMRd: *P* < 2.2e-16, POLE: *P* 8.23E-06, APOBEC: *P* 2.81E-10 and SBS8 *P* < 2.2e-16; Fisher exact test). The contribution values of each signature are shown by a color scale. Color codes representing each dominant mutational signature are shown. **b** TMB of SBS, ID and DBS signatures for UCEC tumors. TMB is measured in somatic mutations per Megabase (Mb). In the TMB plots, columns represent the detected mutational signatures and are ordered by mean somatic mutations per Mb from the lowest frequency, left, to the highest frequency, right. Numbers at the bottom of the TMB plots represent the numbers of tumors harboring each mutational signature. Only samples with counts more than zero are shown. **c** Interaction of signatures with each other. Also see Additional file 3: Table S2 for *P* values calculated by Hypergeometric test. **d** Ternary relation between MMRd, APOBEC and HRd mutational signatures. The ternary plot depicts the contribution of these three signatures as positions in an equilateral triangle. The contribution values of each signature are shown by a color scale. The size of circles represents the prevalence of each signature

The other detected signatures included SBS8 in 25%, APOBEC (SBS2,13) in 39%, and POLE (SBS10a, b) in 10% of tumors, with average contributions of 0.26 (range: 0.15–0.35), 0.23 (range: 0.04–0.81), and 0.28 (range: 0.11–0.88), respectively. Only in one tumor, we detected the MSI/POLE (SBS14) signature with the highest TMB. We also detected the ID1, ID2, ID3, ID7 and ID8 signatures in UCEC whole genomes. The etiology of the D7 and ID8 signatures are associated with MMRd and non-homologous end joining (NHEJ), respectively; they were the first/second dominant signatures in 23% and 53% of UCEC tumors respectively. Finally, we detected DBS2, DBS4, DBS6, DBS9, and DBS11 whose etiologies with the exception of DBS11’s association with APOBEC, remain unknown. DBS11 was the first or second dominant signatures in 21% of UCEC tumors (Fig. 1 and Additional file 1: Fig. S1).

The presence and contribution of detected signatures enabled us to distinguish five tumor groups with distinct patterns of mutational signatures. The dominant signatures were significantly associated with their corresponding signature group. (Fig. 1 and Additional file 1: Fig. S1). MMRd and HRd signatures occurred in a mutually exclusive manner with no overlap between the groups. Like HRd, the APOBEC signatures were mutually exclusive with MMRd. In contrast, ID7 and ID8 were mutually exclusive with HRd (SBS3) as well as DBS2, 4, and 6. However, ID7 co-occurred with the MMRd SBS signatures. DBS2, 4 and 6 co-occurred with SBS8, but were exclusive with the MMRd SBS and ID1, 2, 3, 7, and 8 signatures. Finally, DBS9 was mutually exclusive with the APOBEC SBS signatures. The group of tumors with a dominant POLE signature exhibited other mutational signatures the least; other than POLE, these tumors only had the ID1, 3, 8, and DBS9 signatures. These interactions revealed a ternary relationship between MMRd, HRd, and APOBEC, which respectively had the highest to the lowest prevalence in UCEC (Fig. 1 and Additional file 1: Fig. S1, Additional file 3: Table S2).

**Fig. 2:**
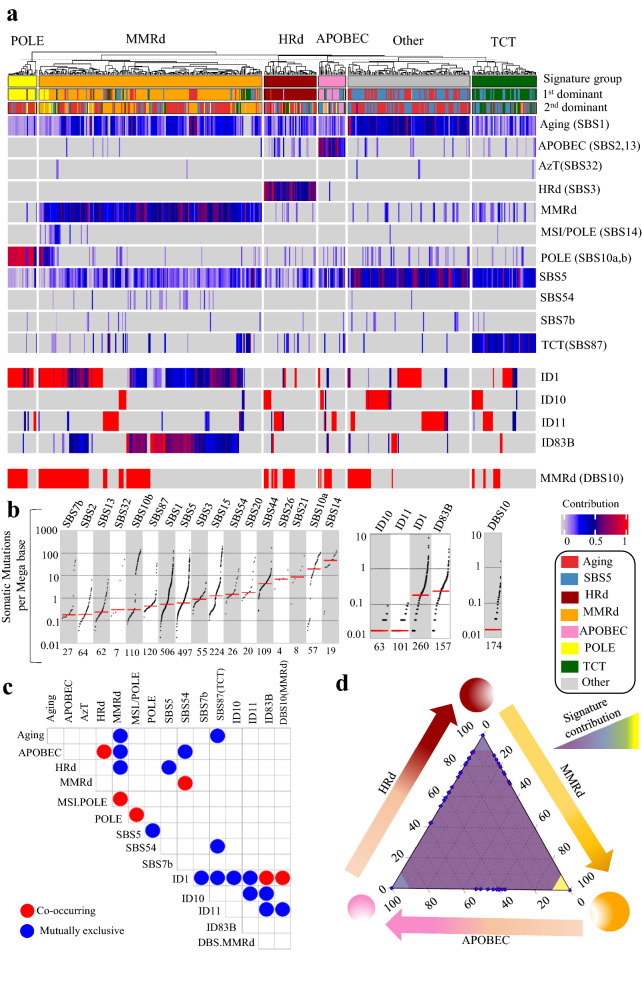
Mutational signature analysis of UCEC WES tumors. **a** NMF-based de novo Mutational signatures of UCEC tumors visualized by a heatmap divided based on major signature status. The first and second dominant signatures are annotated on the top. The dominant signatures were significantly associated with their corresponding signature group (*P* < 2.2e-16, Fisher exact test). The contribution values of each signature are shown by a color scale. Color codes representing each dominant mutational signature are shown. **b** TMB of SBS, ID and DBS signatures for UCEC tumors. TMB is measured in somatic mutations per Megabase (Mb). In the TMB plots, columns represent the detected mutational signatures and are ordered by mean somatic mutations per Mb from the lowest frequency, left, to the highest frequency, right. Numbers at the bottom of the TMB plots represent the numbers of tumors harboring each mutational signature. Only samples with counts more than zero are shown. **c** Interaction of signatures with each other. Also see Additional file 3: Table S3 for *P* values calculated by Hypergeometric test. **d** Ternary relation between MMRd, APOBEC and HRd mutational signatures. The ternary plot depicts the contribution of these three signatures as positions in an equilateral triangle. The contribution values of each signature are shown by a color scale. The size of circles represents the prevalence of each signature

We also analyzed 740,535 SBSs, 8,265 IDs, and 653 DBSs detected in 531 UCEC exomes. The mutational signatures were consistent with those detected in UCEC whole genomes. The prevalence of mutational signatures and their contribution to UCEC tumors were comparable in both WGS and WES analyses, except for the MMRd signatures, SBS21, SBS26, SBS15, and SBS44, which were detected in 53% of the exomes vs. 23% of the whole genomes. Consistent with WGS analysis, MMRd and HRd signatures occurred in a mutually exclusive manner, and APOBEC co-occurred with HRd and was mutually exclusive with MMRd. A group of tumors (23%) showed SBS87 signature which was not detected by WGS data. The proposed etiology of SBS87 is thiopurine chemotherapy treatment (TCT) [40]; it was the dominant signature in 12% of cases with an average contribution value of 0.36 (range: 0.09–0.78). None of the samples in tumors with the TCT signature had HRd (Fig. 2 and Additional file 1: Fig. S2, Additional file 3: Table S3).

In the UCEC exomes, we detected ID1, ID10, and ID11 in addition to a de novo signature, ID83B, which had cosine similarity of 0.42 with COSMIC’s ID1. Among these signatures, only ID1 was detected by both WGS and WES. The only DBS signature detected in the exomes was DBS10 with an etiology associated with MMRd (Fig. 2 and Additional file 1: Fig. S2, Additional file 3: Table S3).

Finally, as a confirmatory approach we performed multivariate analysis for extracting SBS mutational signatures of the UCEC whole genomes and exomes. The data were consistent with those of NMF-based signature extraction. Multivariate analysis also confirmed the pattern of co-occurrence and exclusiveness among HRd, APOBEC and MMRd. Notably, SBS3 and SBS8 co-occurred in UCEC whole genomes (Additional file 1: Fig. S3, Additional file 3: Table S4).

### Mutational signatures of ovarian tumors’ genomes and exomes reveal dominance of HRd signature and co-occurance of HRd with APOBEC

**Fig. 3:**
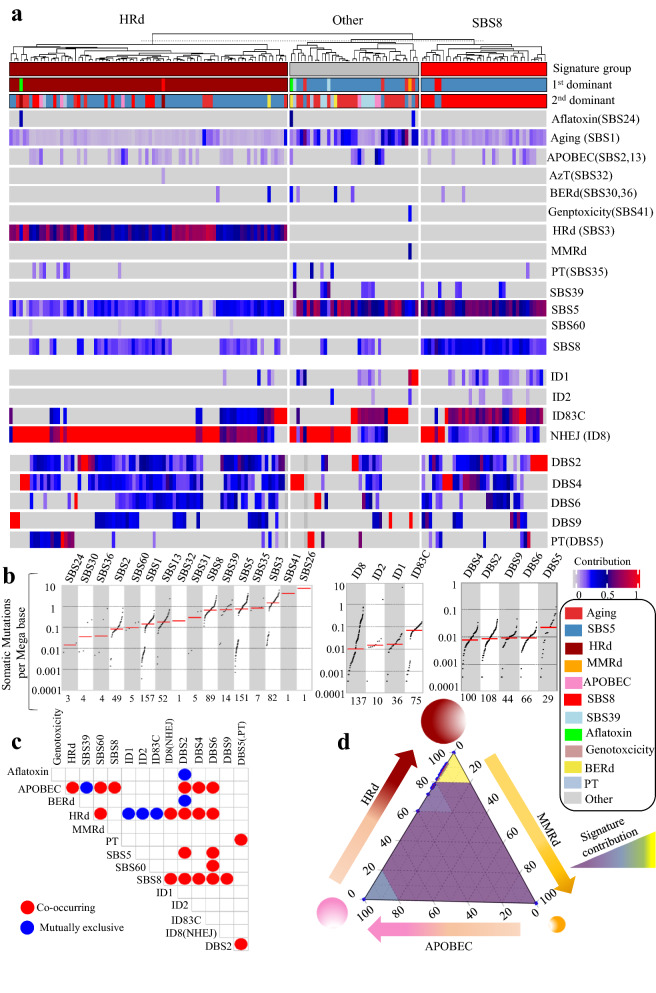
Mutational signature analysis of ovarian WGS tumors. **a** NMF-based de novo mutational signatures of ovarian tumors visualized by a heatmap divided based on major signature status. The First and second dominant signatures are annotated on the top. The dominant signatures were significantly associated with their corresponding signature group (*P* < 2.2e-16, Fisher exact test). The contribution values of each signature are shown by a color scale. Color codes representing each dominant mutational signature are shown. **b** TMB of SBS, ID and DBS signatures for UCEC tumors. TMB is measured in somatic mutations per Megabase (Mb). In the TMB plots, columns represent the detected mutational signatures and are ordered by mean somatic mutations per Mb from the lowest frequency, left, to the highest frequency, right. Numbers at the bottom of the TMB plots represent the numbers of tumors harboring each mutational signature. Only samples with counts more than zero are shown. **c** Interaction of signatures with each other. Also see Additional file 3: Table S5 for *P* values calculated by Hypergeometric test. **d** Ternary relation between MMRd, APOBEC and HRd mutational signatures. The ternary plot depicts the contribution of these three signatures as positions in an equilateral triangle. The contribution values of each signature are shown by a color scale. The size of circles represents the prevalence of each signature

In total, we analyzed 1,180,840 SBSs, 63,073 IDs, and 11,096 DBSs from 157 ovarian tumors’ whole genomes to detect NMF-based de novo mutational signatures. MMRd was present and dominant in only one sample. HRd (SBS3) was present in 52% of tumors in 98% of which it was the first dominant signature. HRd has an average contribution value of 0.55 (range: 0.31–0.86), with the third highest TMB and a median of ~ 1 somatic mutation per Megabase. APOBEC (SBS2, 13) was present in 35% of tumors with an average contribution value of 0.11 (range: 0.04–0.45). APOBEC was never the first dominant and was the second dominant signature in only 4% of tumors. SBS8 was the other major detected signature, present in 57% of tumors with an average contribution value of 0.24 (range: 0.11–0.52). SBS8 was the first or second dominant signature in 31% of tumors. (Fig. 3 and Additional file 1: Fig. S4).

In the whole genomes of ovarian tumors, we detected ID1, ID2, and ID8, in addition to a new signature, ID83C, which had cosine similarity of 0.77 with COSMIC’s ID15, ID2 and ID1. ID1 and ID2’s etiology is associated with replication slippage, and ID8’s is NHEJ [17]. ID8 (NHEJ) was observed in 89% and was first or second dominant ID signature in 83% of cases. These tumors also showed combinations of DBS2, DBS4, DBS6, DBS9, and DBS5. DBS5, which is associated with prior platinum therapy (PT), had the highest TMB. (Fig. 3 and Additional file 1: Fig. S4).

In ovarian tumors, the presence and contribution of detected signatures enabled us to distinguish three main tumor groups with distinct patterns of mutational signatures two of which were dominated by HRd (SBS3) and SBS8 (Fig. 3 and Additional file 1: Fig. S4, Additional file 3: Table S5). APOBEC was present across the groups, and along with ID8(NHEJ), co-occurred significantly with both HRd (SBS3) and SBS8. Notably, DBS2, DBS4 and DBS6 also had co-occurrence with APOBEC, HRd, and SBS8. In these tumors, the ternary relationship between MMRd, HRd, and APOBEC was dominated by the latter two with almost no contribution from MMRd.

**Fig. 4:**
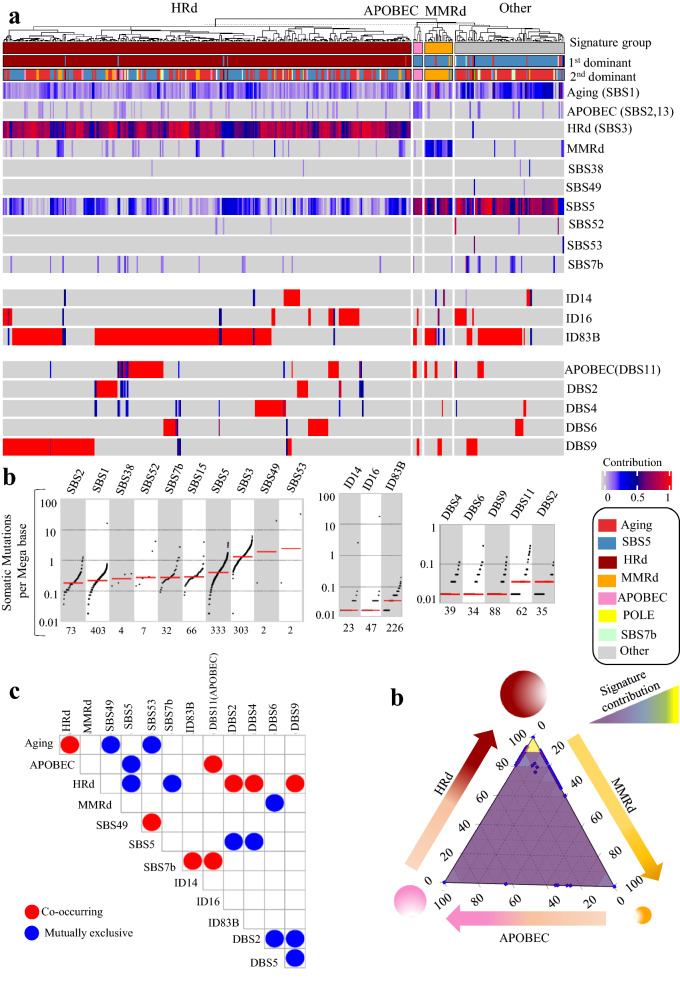
Mutational signature analysis of ovarian WES tumors. **a** NMF-based de novo mutational signatures of ovarian tumors visualized by a heatmap divided based on major signature status. The first and second dominant signatures are annotated on the top. The dominant signatures were significantly associated with their corresponding signature group (HRd and MMRd:* P* < 2.2e-16, APOBEC: *P* 8.52E-11, Fisher exact test). The contribution values of each signature are shown by a color scale. Color codes representing each dominant mutational signature are shown. **b** TMB of SBS, ID and DBS signatures for UCEC tumors. TMB is measured in somatic mutations per Megabase (Mb). In the TMB plots, columns represent the detected mutational signatures and are ordered by mean somatic mutations per Mb from the lowest frequency, left, to the highest frequency, right. Numbers at the bottom of the TMB plots represent the numbers of tumors harboring each mutational signature. Only samples with counts more than zero are shown. **c** Interaction of signatures with each other. Also see Additional file 3: Table S6 for *P* values calculated by Hypergeometric test. **d** Ternary relation between MMRd, APOBEC and HRd mutational signatures. The ternary plot depicts the contribution of these three signatures as positions in an equilateral triangle. The contribution values of each signature are shown by a color scale. The size of circles represents the prevalence of each signature

We also analyzed 47,902 SBSs, 1,796 IDs, and 547 DBSs from 411 ovarian tumor exomes. The mutational signatures were consistent with those detected in the whole genomes. In contrast, to detecting MMRd in only one whole genome, we detected the MMRd-associated SBS15 signature in 16% of the exomes; it was the first or second dominant signature in 5% of tumors with as average contribution of 0.19 (range: 0.07–0.41). HRd (SBS3) was present and was the first or second dominant signature in 74% of tumors with an average contribution value of 0.69 (range: 0.28–1); it had the third highest TMB with a median of ~ 1 somatic mutation per Megabase. APOBEC (SBS2, 13) was present among 18% of tumors, with an average contribution of 0.10 (range: 0.05–0.20). APOBEC was never the first dominant and was the second dominant signature in only 2% of cases (Fig. 4 and Additional file 1: Fig. S5).

We detected ID14, ID16 with unknown etiologies in ovarian tumor exomes, along with ID83B, which had cosine similarity of 0.18 with COSMIC’s ID7. These signatures were all different from the ID signature profile detected in ovarian tumor whole genomes. The exomes also showed DBS2, DBS4, DBS6, DBS9, and DBS11(APOBEC) among which DBS2 and DBS4 co-occurred with HRd (SBS3) (Fig. 4 and Additional file 1: Fig. S5, Additional file 3: Table S6).

Finally, as a confirmatory approach we performed multivariate analysis for extracting SBS mutational signatures of the ovarian tumors’ whole genomes and exomes. The data were consistent with those of NMF-based signature extraction. Notably, SBS3 and SBS8 had co-occurred in ovarian tumors’ whole genomes (Additional file 1: Fig. S6, Additional file 3: Table S7).

### Mutational signatures of cervical tumors’ genomes and exomes reveal dominance of APOBEC signature and its mutual exclusivity with MMRd

Only 20 cervical tumors had available WGS data. In these tumors, we detected three groups based on the first or second dominant detected signature (Additional file 1: Fig. S7 and Additional file 1: Fig. S8). APOBEC was the most prevalent, detected in 65% of cases followed by POLE present in 20%. The remaining samples had SBS5/Aging as the first or second dominant signature.

**Fig. 5:**
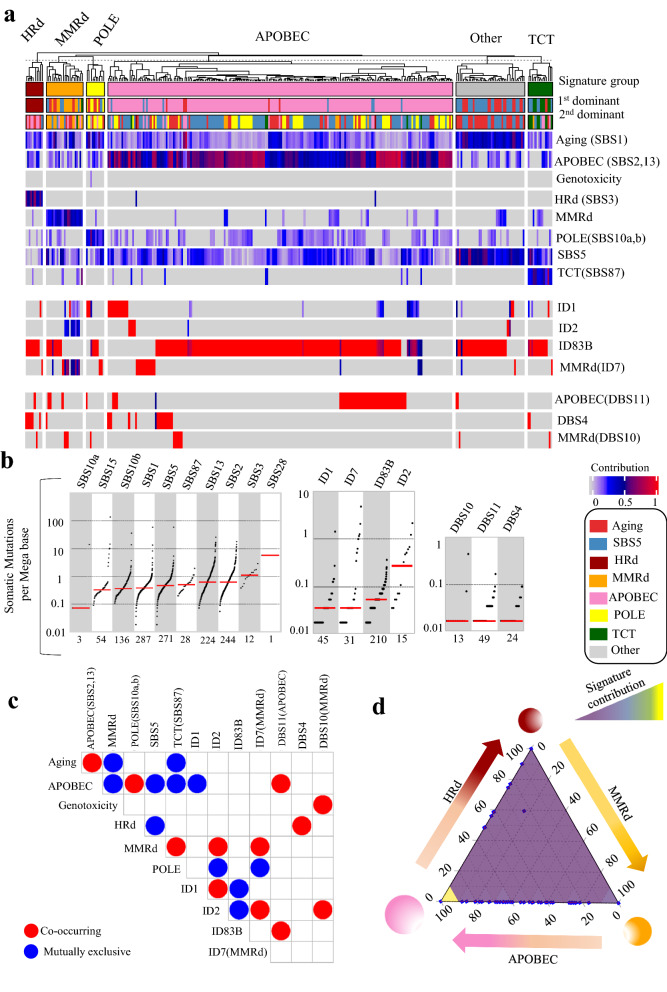
Mutational signature analysis of cervical WES tumors. **a** NMF-based de novo mutational signatures of cervical tumors by a heatmap divided based on major signature status. The first and second dominant signatures are annotated on the top. The dominant signatures were significantly associated with their corresponding signature group (HRd: 2.00E-13, MMRd, APOBEC, TCT:* P* < 2.2e-16, POLE: *P* 1.83E-09, Fisher exact test). The contribution values of each signature are shown by a color scale. Color codes representing each dominant mutational signature are shown. **b** TMB of SBS, ID and DBS signatures for UCEC tumors. TMB is measured in somatic mutations per Megabase (Mb). In the TMB plots, columns represent the detected mutational signatures and are ordered by mean somatic mutations per Mb from the lowest frequency, left, to the highest frequency, right. Numbers at the bottom of the TMB plots represent the numbers of tumors harboring each mutational signature. Only samples with counts more than zero are shown. **c** Interaction of signatures with each other. Also see Additional file 3: Table S8 for *P* values calculated by Hypergeometric test. **d** Ternary relation between MMRd, APOBEC and HRd mutational signatures. The ternary plot depicts the contribution of these three signatures as positions in an equilateral triangle. The contribution values of each signature are shown by a color scale. The size of circles represents the prevalence of each signature

Using WES data from a larger cohort of 289 cervical tumors, we analyzed 81,126 SBSs, 1,929 IDs, and 164 DBSs to detect NMF-based de novo mutational signatures. The MMRd signature, included only SBS15 and was present in 19% of tumors with an average contribution of 0.26 (range: 0.07–0.64). MMRd was the first or second dominant signature among 9% of cases. HRd (SBS3) was present and the first dominant signature in 4% of tumors with an average contribution value of 0.50 (range: 0.39–0.68). APOBEC (SBS2, 13) was present among 85% of tumors with an average contribution of 0.49 (range: 0.08–0.91). APOBEC was first or second dominant signature among 68% of cases. Other major detected signatures, POLE (SBS10a, b) and TCT (SBS87), were present among 48% and 10% of tumors with average contributions of 0.17 (range: 0.08–0.64) and 0.32 (range: 0.13–0.75), and the first or second dominance in 19% and 7% of cases, respectively. Similar to UCEC, the TCT signature was only detected in the exomes and not in the whole genomes of cervical tumors (Fig. 5 and Additional file 1: Fig. S9).

In cervical tumor exomes, we detected ID1, ID2, and ID7 (MMRd), in addition to a de novo signature, ID83B, which had cosine similarity of 0.78 with COSMIC’s ID8 and ID3, and was present in 81% of cases. These tumors also showed DBS4, DBS10 (MMRd) and DBS11(APOBEC) (Fig. 5 and Additional file 1: Fig. S9).

We identified six cervical tumor groups with distinct patterns of mutational signatures. APOBEC (SBS2, 13) was the most prevalent signature; it co-occurred significantly with POLE (SBS10a, b) and APOBEC (DBS11) and was mutually exclusive with the MMRd SBS signatures. MMRd and HRd signature distinguished tumor groups in which APOBEC was also present in a dispersed pattern. The MMRd SBS signatures co-occurred with TCT (SBS87) (Fig. 5 and Additional file 3: Table S8).

Finally, similar to the analyses of the UCEC and ovarian tumors, a multivariate analysis for extracting SBS mutational signatures, confirmed the results from NMF-based signature extraction in the exomes and whole genomes of cervical tumors (Additional file 1: Fig. S10).

### Comparing clinical survival of patients with APOBEC, MMRd and HRd signatures

We compared 5-year OS of patients stratified by mutational signatures suffering from UCEC, ovarian and cervical cancers. In UCEC tumors, the group of patients with HRd and APPBEC had significantly worse survival than the group with MMRd signature (*P* = 0.0037) (Additional file 1: Fig. S11). In ovarian tumors, patients with HRd, APOBEC and MMRd signatures together showed better survival compared to the rest of samples (*P* = 0.0015) (Additional file 1: Fig. S12). In cervical tumors, no significance difference was observed, probably due to limited number of samples in each group restricting the statistical power (Additional file 1: Fig. S13). Finally, we compared survival of patients stratified based on their mutational signatures, independent from their original diagnosis. The results showed that patients with HRd had the poorer outcome followed by APOBEC compared to those with MMRd signature (*P* = 0.016, 0.0001 and 0.0077) (Additional file 1: Fig. S14).

### Mutational signatures gynecological cell lines reflect those seen in primary tumors

We analyzed 106,917 SBSs, 10,925 IDs, and 135 DBSs detected by WES in 40 uterine cell lines. The MMRd signatures, including SBS6, SBS20, and SBS26, were present in 24 cell lines with an average contribution value of 0.71. When present, MMRd was the first or second dominant signature. POLE (SBS10a, b) was present in four cell lines in three of which it was the first dominant signature; it had an average contribution of was 0.55. Moreover, DBS11 (APOBEC) was present and dominant in 19 cell lines with an average contribution of 0.98.

For the MMRd and POLE SBS signatures, the results from multivariate analysis were consistent with the signature extracted using the NMF-based approach. However, HRd and APOBEC were only detected by multivariate analysis of cell lines. HRd (SBS3) was present in 12 cell lines with an average contribution of 0.07. HRd was the second dominant signature in two cell lines. Finally, APOBEC was present in 21 cell lines with an average contribution of 0.05; it was the second dominant signature in two cell lines (Additional file 1: Fig. S15).

Next, we analyzed 43,757 SBSs, 4,717 IDs, and 134 DBSs, detected in the exomes of 74 ovarian cell lines. The MMRd signatures, including SBS15 and SBS44, were present in 38 cell lines with an average contribution of 0.24. MMRd was the first or second dominant signature in 15 cell lines. HRd (SBS3) was present among 18 cell lines with an average contribution value of 0.42; it was the first or second dominant signature when present. POLE (SBS10a, b) was present in 8 cell lines with an average contribution value of was 0.14; it was the first dominant signature in only one tumor. DBS11 (APOBEC) was present and dominant in 22 cell lines with the average contribution value of 0.95. Multivariate analysis was consistent with NMF-based signature extraction (Additional file 1: Fig. S16, Additional file 3: Table S8).

Finally, we analyzed 10,842 SBSs, 725 IDs, and 47 DBSs detected in 21 cervical cell line exomes. The MMRd signatures, including SBS6, and SBS26 were present in four cell lines with an average contribution of 0.34. MMRd was the first or second dominant signature in three cell lines. APOBEC was present in 15 cell lines with an average contribution value of 0.31; it was the first or second dominant signature in 12 cell lines. Multivariate analysis did not detect MMRd; however, it found HRd(SBS3) present in 11 cell lines with an average contribution value of 0.1. HRd was second dominant signature in one cell line. Multivariate analysis also detected APOBEC in 20 cell lines with an average contribution value of 0.27; APOBEC was first/second dominant signature in 14 cell lines (Additional file 1: Fig. S17, Additional file 3: Table S8).

## Discussion

In this work, we present the identification of distinct patterns of interaction between mutational signatures across gynecological cancers, suggesting possible precision therapeutic targets. Our findings provide evidence for mutual exclusivity of MMRd and HRd in all gynecological tumors, which is consistent with our unpublished data on colorectal and stomach cancers. Co-occurrence of the APOBEC and HRd signatures indicates positive interactions between cooperating mutational processes which together suggests therapeutic vulnerabilities with important implications for the development of new personalize therapies for these cancers. Considering presence of inter- and intra-individual heterogeneity in mutational signatures, the selection of appropriate combination therapies based on a tumor’s signature profile may help prevent emergence of therapeutic resistance.

Until recently, mutational signatures were typically characterized by SBSs; however, mutational processes in human somatic cells are not limited to base substitutions [17], and DNA damage and defected DNA repair processes may result in small insertion and deletions or large-scale chromosomal aberrations and structural variation [5]. In addition to SBS, we comprehensively profiled ID and DBS signatures. Co-occurrence of DBS2, DBS4, and DBS6 with HRd and/or APOBEC suggests possible related etiology for them.

NMF-based methods have difficulty inferring some signatures such as SBS3, 5, 8, and 40 [17, 41]; thus, we confirmed our results by a complimentary approach based on multivariate analysis. Notably, multivariate analysis showed that SBS3 and SBS8 co-occurred in UCEC and ovarian tumor whole genomes. APOBEC and HRd were detected at a higher rate in the whole genomes compared to the exomes, in contrast to MMRd and POLE which had higher prevalence in the exomes. These results suggest that MMRd may preferentially occur in regions probed by WES. Conversely, HRd and APOBEC may be present in a dispersed pattern across the entire genome. While ID1was detected in both WES and WGS data, it had a higher prevalence in WES regions.

Tumors with predominant MMRd signature are potential candidates for treatment with immune checkpoint inhibitors such as pembrolizumab [16]. We confirmed presence of the MMRd signature in subsets of tumors in all gynecological cancer types, with the highest prevalence in UCEC, following by cervical and ovarian tumors. Similarly, detection of HRd in gynecological tumors provides a potentially actionable biomarker that suggests possible efficacy of PARPi therapy, immune checkpoint blockade therapy, and/or DNA-damaging chemotherapy for treatment of these tumors.

APOBEC is one of the most prominent mutation signatures in cancer, present in over half of human tumors [14, 42]. APOBEC signature was present across all three gynecological tumor types, but it was the most dominant in cervical tumors. APOBEC3-dependent mutagenesis may impose selective pressures on tumor cell and offer opportunities for therapeutic intervention via induction of synthetic lethality [43, 44]. Put together, these results highlight the need for integration of signature-based biomarkers in treatment decisions. Patient stratification based on mutational signature exposure can identify patients who are potentially suitable candidates for targeted therapy.

We also inferred mutational signature profiles in gynecological cell lines. Our results propose available cell line with mutational signatures characterized in primary tumors; these cell lines can serve as experimental models for mechanistic and therapeutic studies. Although multiple UCEC cell lines present MMRd; there are no appropriate cell lines available with the HRd and APOBEC signatures. Similarly, MMRd and HRd are detected in several ovarian cancer cell lines; however, we did not identify any cell lines representing the APOBEC signature. In contrast, there are several cervical cancer cell lines with APOBEC as a dominant signature; yet, only three present MMRd and none exist with HRd. These findings underscore the need for developing related model cell lines that fully capture mutational signature profile of primary gynecological tumors.

Large numbers of somatic mutations accrue in human cancers; however, the underlying causes for most of them remains largely unknown. Identifying driver mutations has been one of the main goals in cancer research; the attention is now turning to studying mutational imprints of DNA damage and defected DNA repair processes that are embedded in the entire somatic profiles across the coding and non-coding regions. Accurately capturing mutational processes operating during tumorigenesis using mutational signature analysis will not only provide insights on their mechanistic basis and the biological history of a tumor; but also it may help to prevent patients from receiving incorrect treatment or missing out on possible treatment opportunities.

## Conclusion

This study provides a comprehensive profile of mutational signatures based on SBS, ID and DBS mutations in gynecological malignancies using two complementary approaches. Our results show the prevalence and contribution of mutational signatures, which vary among gynecological cancer types, and reveal a consistent tumor type-independent ternary relation between MMRd, HRd and APOBEC where HRd is mutually exclusive with MMRd, and APOBEC co-occurrs with HRd and while being mutually exclusive with MMRd.

## Supplementary Information

**Additional file 1.** Supplementary figures and information include Fig S1 ~ Fig. S17.

**Additional file 2.** Supplementary tables (Table S1 A~X) provide results of mutational signature analysis by NMF-based and multivariate approach.

**Additional file 3.** Table S2~S9. P values of mutational signature interactions.

## Data Availability

All data generated or analyzed during this study are included in this published article and its supplementary information files. All in-house R scripts used for the analyses are available upon request.

## Abbreviations

MMRd: Mismatch repair deficiency
HRd: Homologous recombination deficiency
MSI: Microsatellite instability
PARPi: Poly (ADP-ribose) polymerase inhibitors
APOBEC: Apolipoprotein B mRNA editing enzyme, Catalytic polypeptide-like
ROS: Reactive oxygen species.
PT: Platinum therapy
TCT: Thiopurine chemotherapy treatment
NHEJ: Non-homologous end joining
ICGC: International Cancer Genome Consortium
COSMIC: Catalogue of Somatic Mutations in Cancer
CCLE: Cancer Cell Line Encyclopedia
Mb: Megabase
IDs: Insertions/Deletions
SBSs: Single-Base Substitutions
DBSs: Double-Base Substitutions
SSM: Simple Somatic Mutation
WES: Whole exome sequencing
WGS: Whole genome sequencing
NMF: Non-Negative Matrix Factorization
TMB: Tumor mutational burden
UCEC: Uterine corpus endometrial carcinoma
OS: Overall Survival
