# Supplementary material for: Mutational signatures reveal ternary relationships between homologous recombination repair, APOBEC, and mismatch repair in gynecological cancers
Source: J Transl Med. 2022 Feb 2;20:65. doi: 10.1186/s12967-022-03259-0 (PMC8812249; doi:10.1186/s12967-022-03259-0)
Supplement: Supplementary file 1 — Additional file 1. Supplementary figures and information include Fig S1 ~ Fig. S17. [file 12967_2022_3259_MOESM1_ESM.pdf]

Additional File 1

Mutational signatures reveal ternary relationships between homologous recombination repair, APOBEC and mismatch repair in gynecological cancers

Amir Farmanbar[1*], Sanaz Firouzi[2*#], Robert Kneller[3], Hossein Khiabanian[1,4#]

[1] Center for Systems and Computational Biology, Rutgers Cancer Institute of New Jersey, Rutgers University, New Brunswick, NJ, 08903, USA

[2]Institute for Cancer Genetics, Columbia University, New York, NY 10032, USA

[3]Research Center for Advanced Science and Technology, University of Tokyo, Minato-ku, Tokyo, 153-8904, Japan

[4]Department of Pathology and Laboratory Medicine, Rutgers Robert Wood Johnson Medical School, Rutgers University, New Brunswick, NJ, 08903, USA

SUPPLEMENTARY FIGURE LEGENDS

Fig. S1: Dominant signature analysis of UCEC whole genomes. **(a)** The first and second dominant SBS mutational signatures (**b)** The first and second dominant ID mutational signatures (**c)** The first and second dominant DBS mutational signatures Dominant signatures were based on the contribution value of detected mutational signatures. For mutational signatures with known etiology, both signature and etiology are indicated.

Fig. S2: Dominant signature analysis of UCEC exomes. **(a)** The first and second dominant SBS mutational signatures (**b)** The first and second dominant ID mutational signatures (**c)** The first and second dominant DBS mutational signatures Dominant signatures were based on the contribution value of detected mutational signatures. For mutational signatures with known etiology, both signature and etiology are indicated.

Fig. S3: Mutational signature analysis of UCEC whole genomes and exomes by multivariate analysis. (**a**) Mutational signatures of UCEC whole genomes (**b**) Interaction of signatures with each other in UCEC whole genomes. **(c)**Mutational signatures of UCEC exomes. The heatmap is divided based on dominant signature status. The First and second dominant signatures are annotated on the top of each heatmap. The contribution values of each signature are shown by a color scale. Color codes representing each dominant mutational signature are shown.

Fig. S4: Dominant signature analysis of ovarian WGS tumors. **(a)** The first and second dominant SBS mutational signatures (**b)** The first and second dominant ID mutational signatures (**c)** The first and second dominant DBS mutational signatures Dominant signatures were based on the contribution value of detected mutational signatures. For mutational signatures with known etiology, both signature and etiology are indicated.

Fig. S5: Dominant signature analysis of ovarian WES tumors. **(a)** The first and second dominant SBS mutational signatures (**b)** The first and second dominant ID mutational

signatures **(c)** The first and second dominant DBS mutational signatures Dominant signatures were based on the contribution value of detected mutational signatures. For mutational signatures with known etiology, both signature and etiology are indicated.

Fig. S6: Mutational signature analysis of ovarian WGS tumors and exomes by multivariate analysis. (**a)** Mutational signatures of ovarian tumors' whole genomes (**b)** Interaction of signatures with each other in ovarian tumors' whole genomes. **(c)**Mutational signatures of ovarian tumors' exomes. The heatmap is divided based on dominant signature status. The First and second dominant signatures are annotated on the top of each heatmap. The contribution values of each signature are shown by a color scale. Color codes representing each dominant mutational signature are shown.

Fig. S7: Mutational signature analysis of cervical WGS tumors by NMF-based signature extraction. (**a)** Mutational signatures of cervical tumors' whole genomes. The heatmap is divided based on dominant signature status. The First and second dominant signatures are annotated on the top of each heatmap. The contribution values of each signature are shown by a color scale. Color codes representing each dominant mutational signature are shown. **(b)** TMB of SBS, ID and DBS signatures for cervical tumors' whole genomes. TMB is measured in somatic mutations per Megabase (Mb). In the TMB plots, columns represent the detected mutational signatures and are ordered by mean somatic mutations per Mb from the lowest frequency, left, to the highest frequency, right. Numbers at the bottom of the TMB plots represent the numbers of tumors harboring each mutational signature. Only samples with counts more than zero are shown.

Fig. S8: Dominant signature analysis of cervical WGS tumors. (a) The first and second dominant SBS mutational signatures (**b)** The first and second dominant ID mutational signatures (**c)** The first and second dominant DBS mutational signatures Dominant signatures were based on the contribution value of detected mutational signatures. For mutational signatures with known etiology, both signature and etiology are indicated.

Fig. S9: Dominant signature analysis of cervical WES tumors. **(a)** The first and second dominant SBS mutational signatures (**b)** The first and second dominant ID mutational signatures (**c)** The first and second dominant DBS mutational signatures Dominant signatures were based on the contribution value of detected mutational signatures. For mutational signatures with known etiology, both signature and etiology are indicated.

Fig. S10: Mutational signature analysis of cervical WGS tumors and exomes by multivariate analysis. (**a)** Mutational signatures of cervical tumors' whole genomes. **(b)** Mutational signatures of cervical tumors' exomes. The heatmap is divided based on dominant signature status. The First and second dominant signatures are annotated on the top of each heatmap. The contribution values of each signature are shown by a color scale. Color codes representing each dominant mutational signature are shown.

Fig. S11: Comparing survival of patients with APOBEC, HRd and MMRd signatures in UCEC exomes. (**a)** Kaplan-Meyer curves representing OS of patients for all signature groups **(b)** Kaplan-Meyer curves representing OS of patients stratified by harboring MMRd compared to APOBEC and HRd signatures. **(c)** Kaplan-Meyer curves representing OS of patients stratified by harboring MMRd compared to HRd signatures. **(d)** Kaplan-Meyer curves representing OS of patients stratified by harboring MMRd compared to APOBEC signatures. **(e)** Kaplan-Meyer curves representing OS of patients stratified by harboring HRd compared to APOBEC signatures. P values represent the significance determined from log-rank.

Fig. S12: Comparing survival of patients with APOBEC, HRd and MMRd signatures in ovarian tumors' exomes. (**a)** Kaplan-Meyer curves representing OS of patients for all signature groups **(b)** Kaplan-Meyer curves representing OS of patients stratified by harboring APOBEC, HRd, MMRd signatures compared to the rest of samples. **(c)** Kaplan-Meyer curves representing OS of patients stratified by harboring HRd compared to other signature group. **(d)** Kaplan-Meyer curves representing OS of patients stratified by harboring HRd compared to APOBEC signatures. **(e)** Kaplan-Meyer curves

representing OS of patients stratified by harboring HRd compared to MMRd signatures. **(f)** Kaplan-Meyer curves representing OS of patients stratified by harboring MMRd compared to APOBEC signatures. P values represent the significance determined from log-rank.

Fig. S13: Comparing survival of patients with APOBEC, HRd and MMRd signatures in cervical tumors' exomes. (**a)** Kaplan-Meyer curves representing OS of patients for all signature groups **(b)** Kaplan-Meyer curves representing OS of patients stratified by harboring APOBEC compared to HRd signatures **(c)** Kaplan-Meyer curves representing OS of patients stratified by harboring APOBEC compared to MMRd signatures **(d)** Kaplan-Meyer curves representing OS of patients stratified by harboring HRd compared to MMRd signatures. P values represent the significance determined from log-rank.

Fig. S14: Comparing survival of patients with APOBEC, HRd and MMRd signatures in aggregated UCEC, ovarian and cervical tumors' exomes. (**a)** Kaplan-Meyer curves representing OS of patients for all signature groups **(b)** Kaplan-Meyer curves representing OS of patients stratified by harboring APOBEC compared to HRd signatures **(c)** Kaplan-Meyer curves representing OS of patients stratified by harboring APOBEC compared to MMRd signatures **(d)** Kaplan-Meyer curves representing OS of patients stratified by harboring HRd compared to MMRd signatures. P values represent the significance determined from log-rank.

Fig. S15: Mutational signature analysis of uterine cell lines' exomes by NMF-based signature extraction. (**a)** Mutational signatures of uterine cell lines. The heatmap is divided based on dominant signature status. The First and second dominant signatures are annotated on the top of each heatmap. The contribution values of each signature are shown by a color scale. Color codes representing each dominant mutational signature are shown. **(b)** TMB of SBS, ID and DBS signatures. TMB is measured in somatic mutations per Megabase (Mb). In the TMB plots, columns represent the detected mutational

signatures and are ordered by mean somatic mutations per Mb from the lowest frequency, left, to the highest frequency, right. Numbers at the bottom of the TMB plots represent the numbers of tumors harboring each mutational signature. Only samples with counts more than zero are shown. (c) Interaction of signatures with each other. Also see Additional file 3: Table S9 for P values.

Fig. S16: Mutational signature analysis of ovarian cell lines' exomes by NMF-based signature extraction. (**a**) Mutational signatures of ovarian cell lines. The heatmap is divided based on dominant signature status. The First and second dominant signatures are annotated on the top of each heatmap. The contribution values of each signature are shown by a color scale. Color codes representing each dominant mutational signature are shown. **(b)** TMB of SBS, ID and DBS signatures. TMB is measured in somatic mutations per Megabase (Mb). In the TMB plots, columns represent the detected mutational signatures and are ordered by mean somatic mutations per Mb from the lowest frequency, left, to the highest frequency, right. Numbers at the bottom of the TMB plots represent the numbers of tumors harboring each mutational signature. Only samples with counts more than zero are shown. (c) Interaction of signatures with each other. Also see Additional file 3: Table S9 for P values.

Fig. S17: Mutational signature analysis of cervical cell lines' exomes by NMF-based signature extraction. (**a**) Mutational signatures of cervical cell lines. The heatmap is divided based on dominant signature status. The First and second dominant signatures are annotated on the top of each heatmap. The contribution values of each signature are shown by a color scale. Color codes representing each dominant mutational signature are shown. **(b)** TMB of SBS, ID and DBS signatures. TMB is measured in somatic mutations per Megabase (Mb). In the TMB plots, columns represent the detected mutational signatures and are ordered by mean somatic mutations per Mb from the lowest frequency, left, to the highest frequency, right. Numbers at the bottom of the TMB plots represent the numbers of tumors harboring each mutational signature. Only samples with counts more than zero are shown. (c) Interaction of signatures with each other. Also see Additional file 3: Table S9 for P values.

Fig. S1

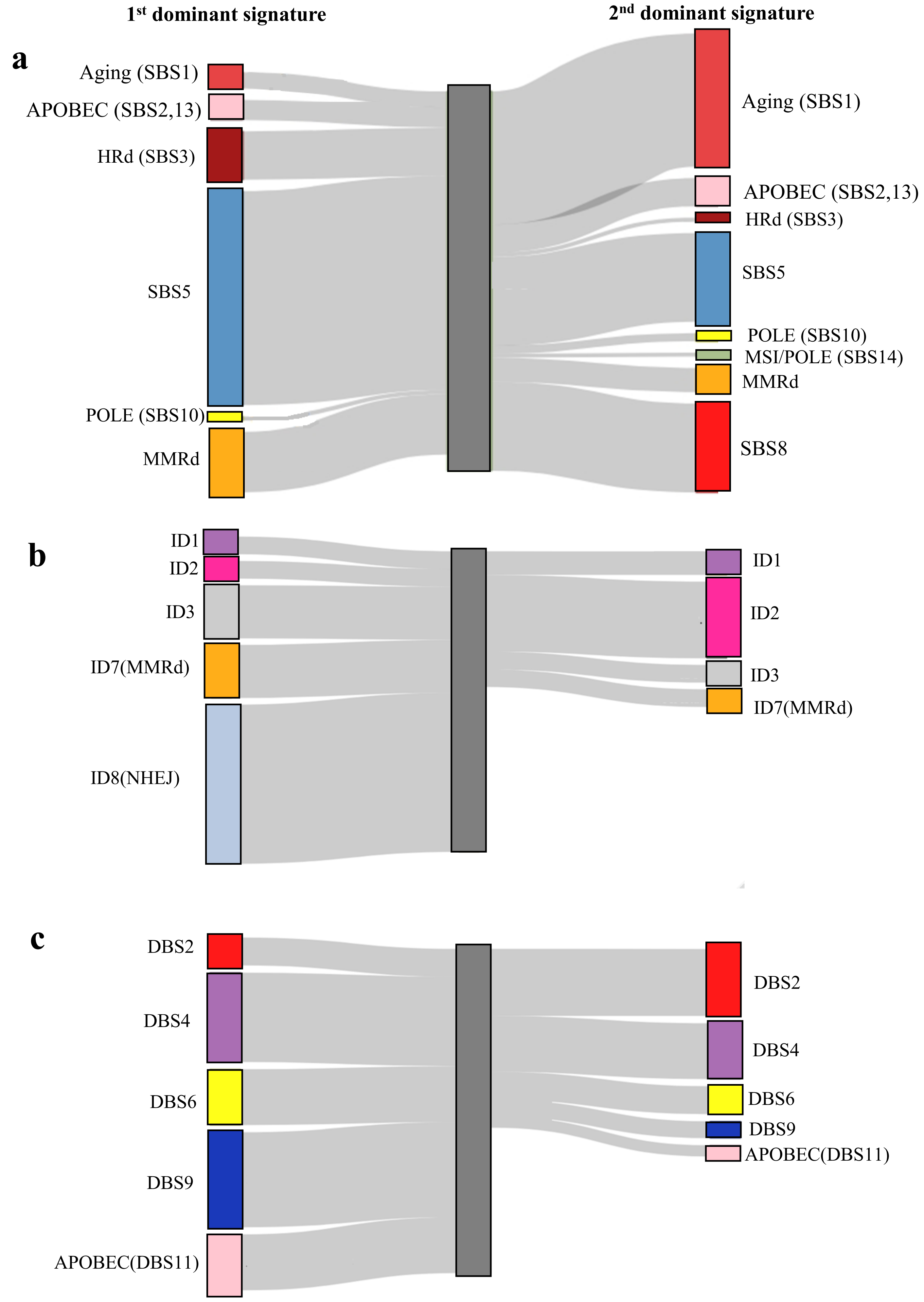
1st dominant signature
2nd dominant signature
a
Aging (SBS1)
APOBEC (SBS2,13)
HRd (SBS3)
SBS5
POLE (SBS10)
MMRd
Aging (SBS1)
APOBEC (SBS2,13)
HRd (SBS3)
SBS5
POLE (SBS10)
MSI/POLE (SBS14)
MMRd
SBS8
b
ID1
ID2
ID3
ID7(MMRd)
ID8(NHEJ)
ID1
ID2
ID3
ID7(MMRd)
c
DBS2
DBS4
DBS6
DBS9
APOBEC(DBS11)
DBS2
DBS4
DBS6
DBS9
APOBEC(DBS11)

Fig. S2

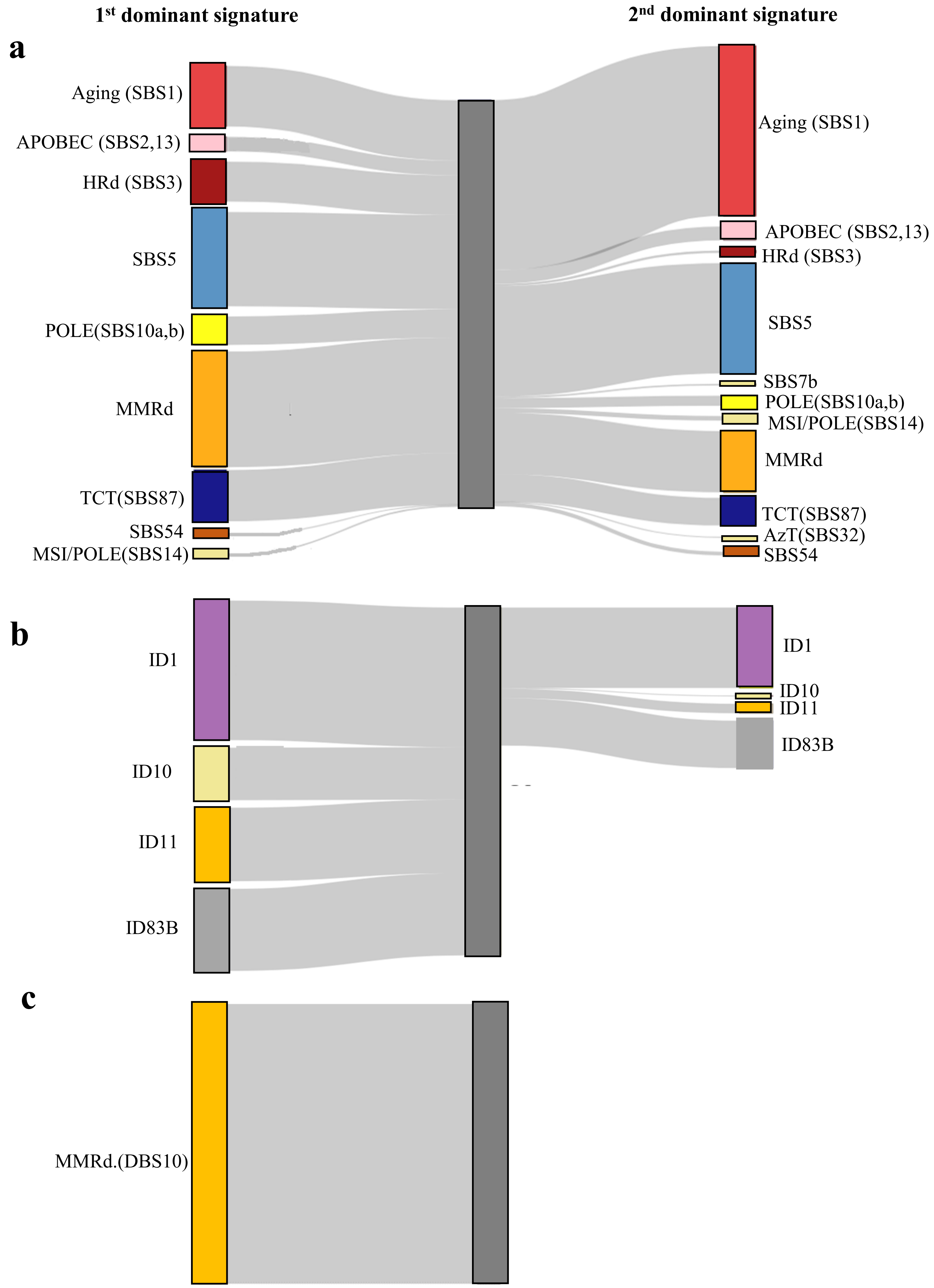
1st dominant signature
2nd dominant signature
a
Aging (SBS1)
APOBEC (SBS2,13)
HRd (SBS3)
SBS5
POLE(SBS10a,b)
MMRd
TCT(SBS87)
SBS54
MSI/POLE(SBS14)
Aging (SBS1)
APOBEC (SBS2,13)
HRd (SBS3)
SBS5
SBS7b
POLE(SBS10a,b)
MSI/POLE(SBS14)
MMRd
TCT(SBS87)
AzT(SBS32)
SBS54
b
ID1
ID10
ID11
ID83B
ID1
ID10
ID11
ID83B
c
MMRd.(DBS10)

Fig. S3

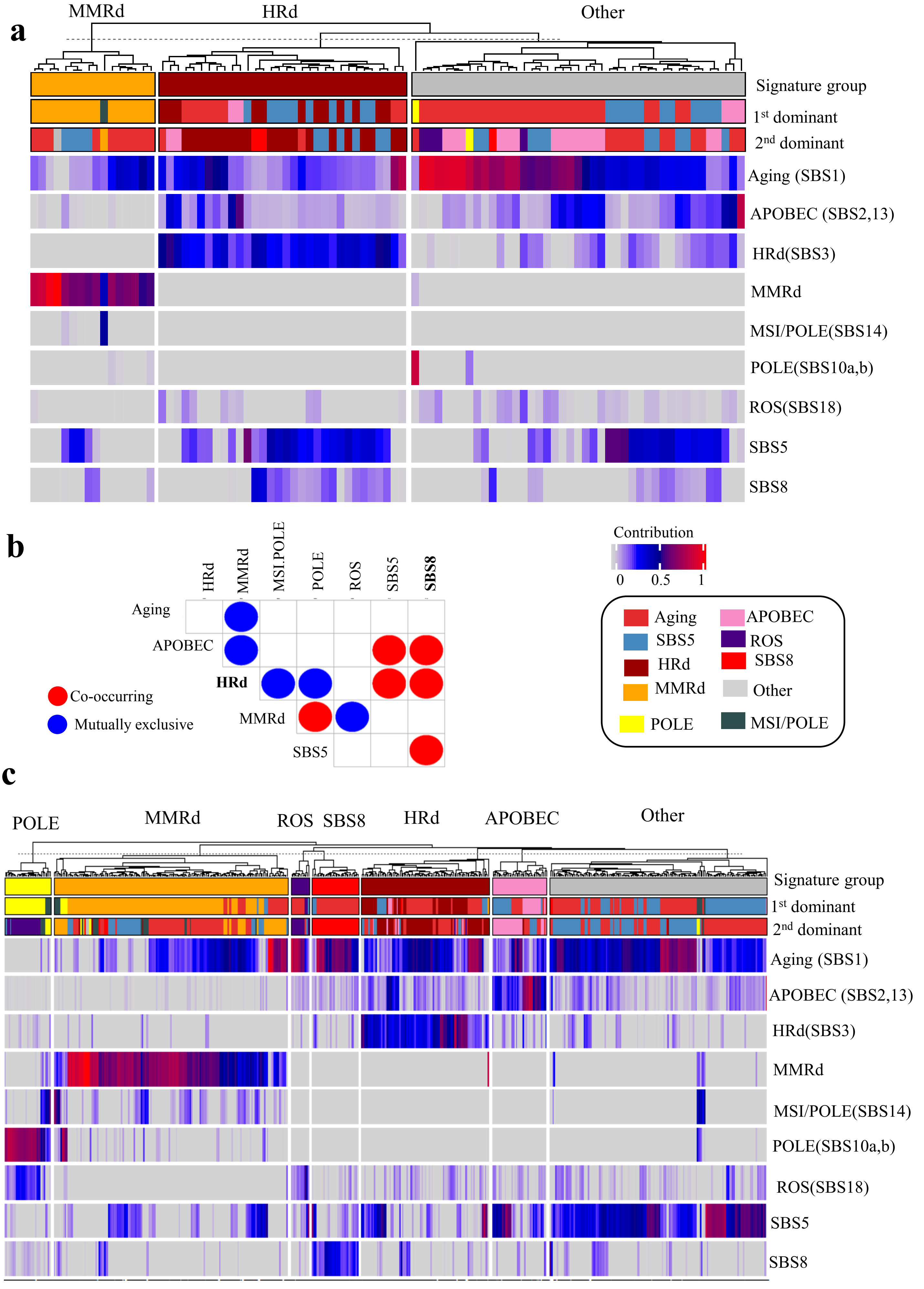

a
MMRd
HRd
Other
Signature group
1st dominant
2nd dominant
Aging (SBS1)
APOBEC (SBS2,13)
HRd(SBS3)
MMRd
MSI/POLE(SBS14)
POLE(SBS10a,b)
ROS(SBS18)
SBS5
SBS8
b
HRd
MMRd
MSI.POLE
POLE
ROS
SBS5
SBS8
Aging
APOBEC
HRd
MMRd
SBS5
Co-occurring
Mutually exclusive
Contribution
0
0.5
1
Aging
APOBEC
SBS5
ROS
HRd
SBS8
MMRd
Other
POLE
MSI/POLE
c
POLE
MMRd
ROS
SBS8
HRd
APOBEC
Other
Signature group
1st dominant
2nd dominant
Aging (SBS1)
APOBEC (SBS2,13)
HRd(SBS3)
MMRd
MSI/POLE(SBS14)
POLE(SBS10a,b)
ROS(SBS18)
SBS5
SBS8

Fig. S4

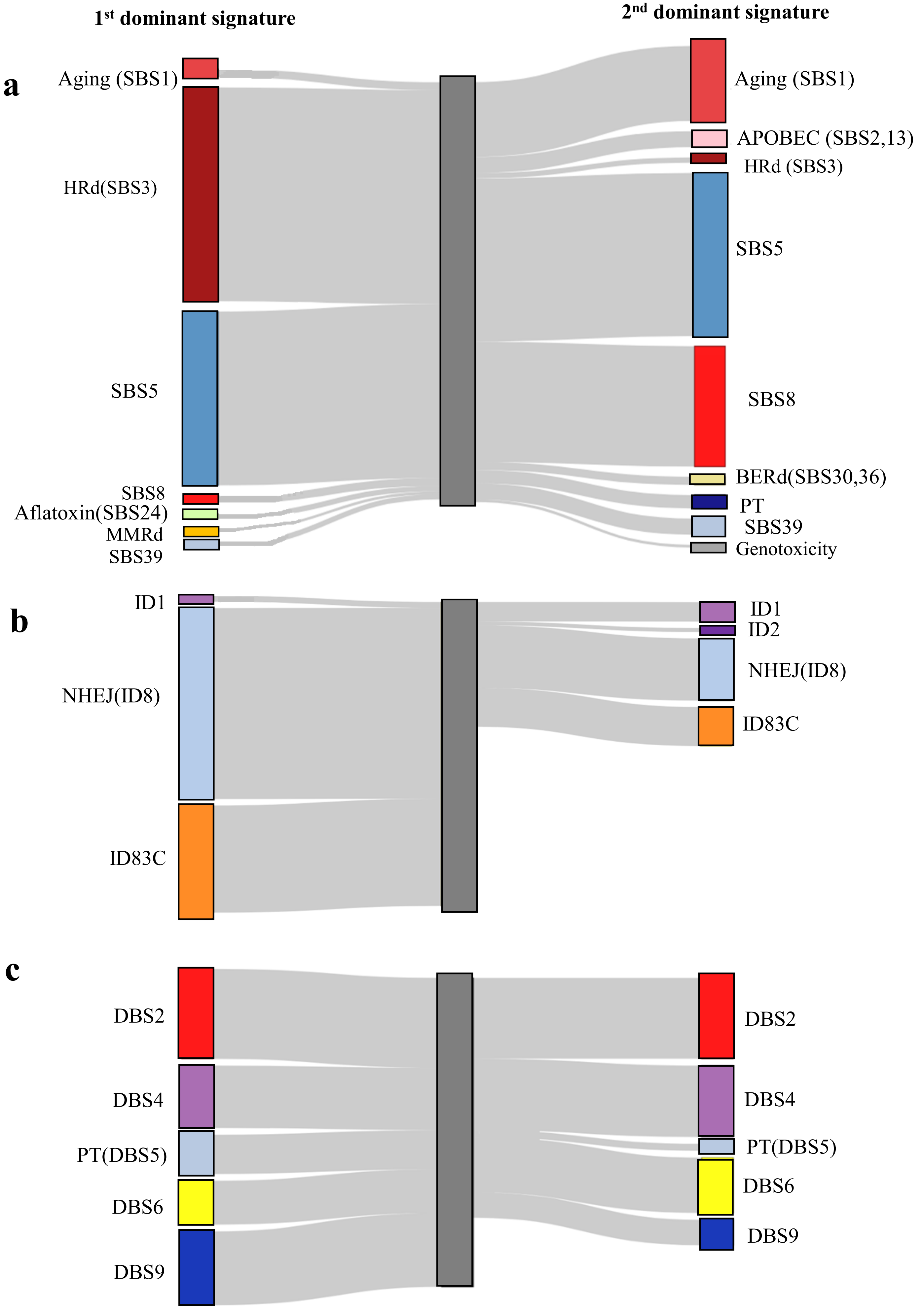

1st dominant signature
2nd dominant signature
a
Aging (SBS1)
HRd(SBS3)
SBS5
SBS8
Aflatoxin(SBS24)
MMRd
SBS39
Aging (SBS1)
APOBEC (SBS2,13)
HRd (SBS3)
SBS5
SBS8
BERd(SBS30,36)
PT
SBS39
Genotoxicity
b
ID1
NHEJ(ID8)
ID83C
ID1
ID2
NHEJ(ID8)
ID83C
c
DBS2
DBS4
PT(DBS5)
DBS6
DBS9
DBS2
DBS4
PT(DBS5)
DBS6
DBS9

Fig. S5

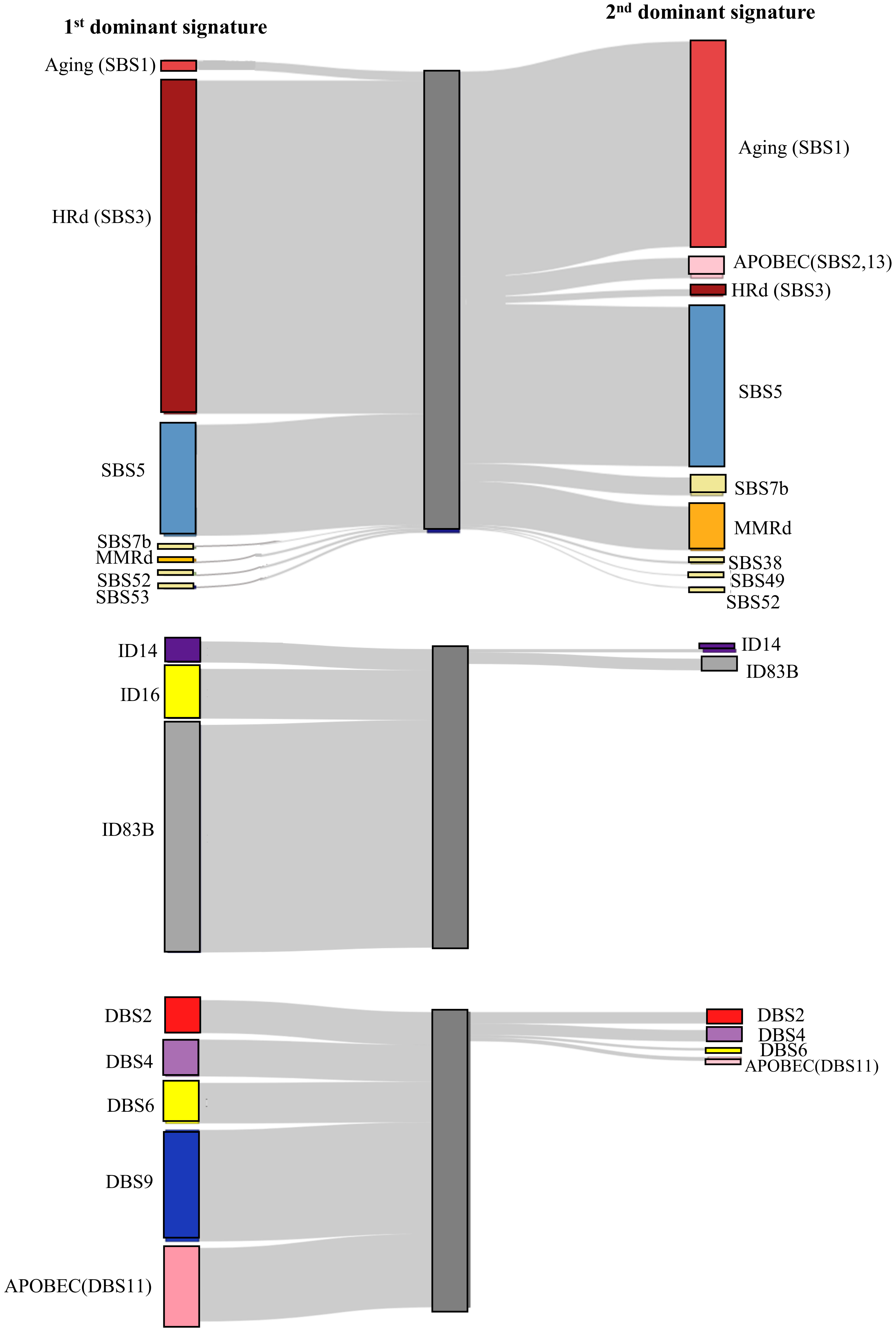

1st dominant signature
2nd dominant signature
Aging (SBS1)
HRd (SBS3)
SBS5
SBS7b
MMRd
SBS52
SBS53
Aging (SBS1)
APOBEC(SBS2,13)
HRd (SBS3)
SBS5
SBS7b
MMRd
SBS38
SBS49
SBS52
ID14
ID16
ID83B
ID14
ID83B
DBS2
DBS4
DBS6
DBS9
APOBEC(DBS11)
DBS2
DBS4
DBS6
APOBEC(DBS11)

Fig. S6

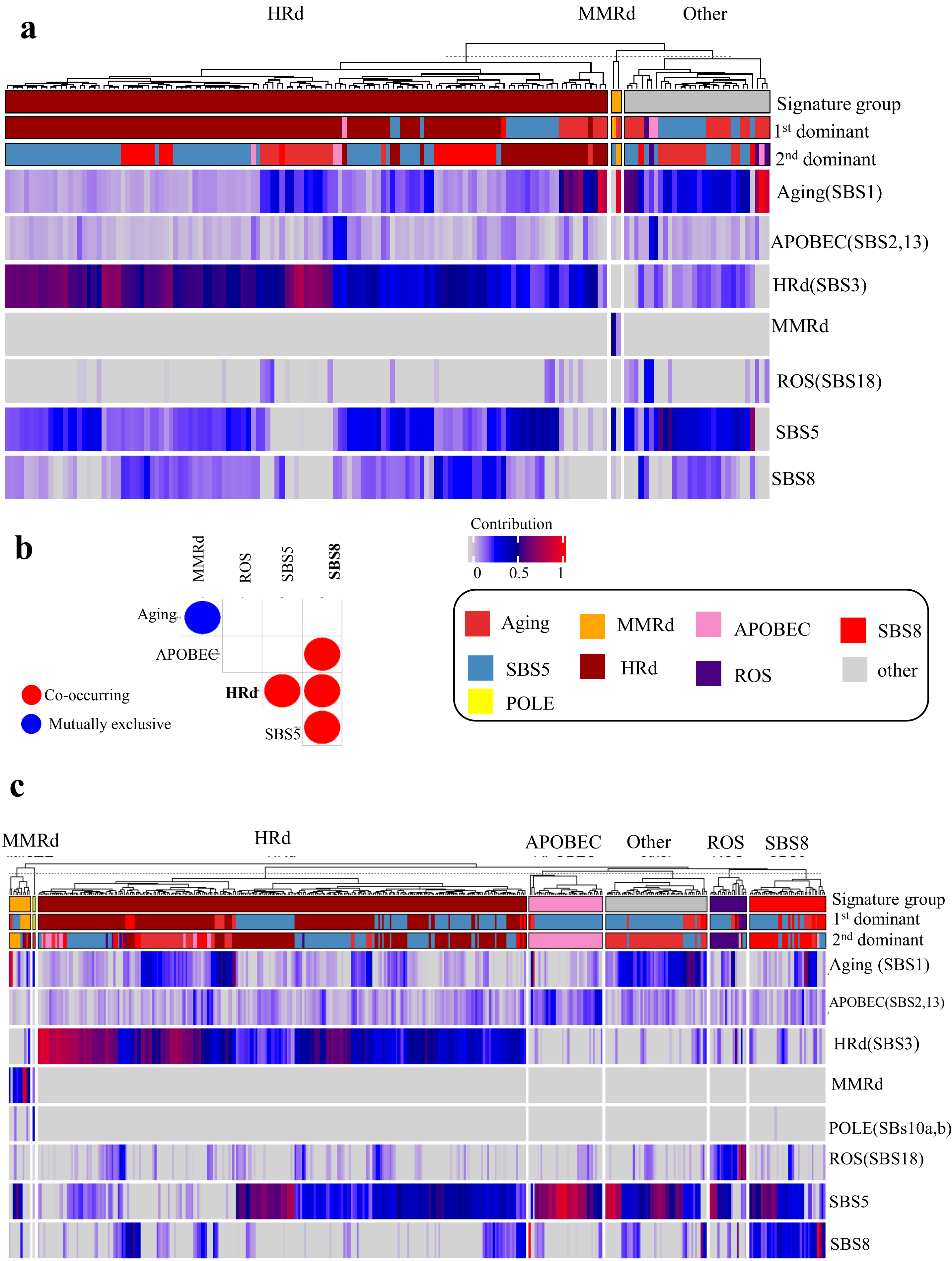
a
HRd
MMRd
Other
Signature group
1st dominant
2nd dominant
Aging(SBS1)
APOBEC(SBS2,13)
HRd(SBS3)
MMRd
ROS(SBS18)
SBS5
SBS8
b
MMRd
ROS
SBS5
SBS8
Aging
APOBEC
HRd
SBS5
Co-occurring
Mutually exclusive
Contribution
0
0.5
1
Aging
MMRd
APOBEC
SBS8
SBS5
HRd
ROS
other
POLE
c
MMRd
HRd
APOBEC
Other
ROS
SBS8
Signature group
1st dominant
2nd dominant
Aging (SBS1)
APOBEC(SBS2,13)
HRd(SBS3)
MMRd
POLE(SBs10a,b)
ROS(SBS18)
SBS5
SBS8

Fig. S7

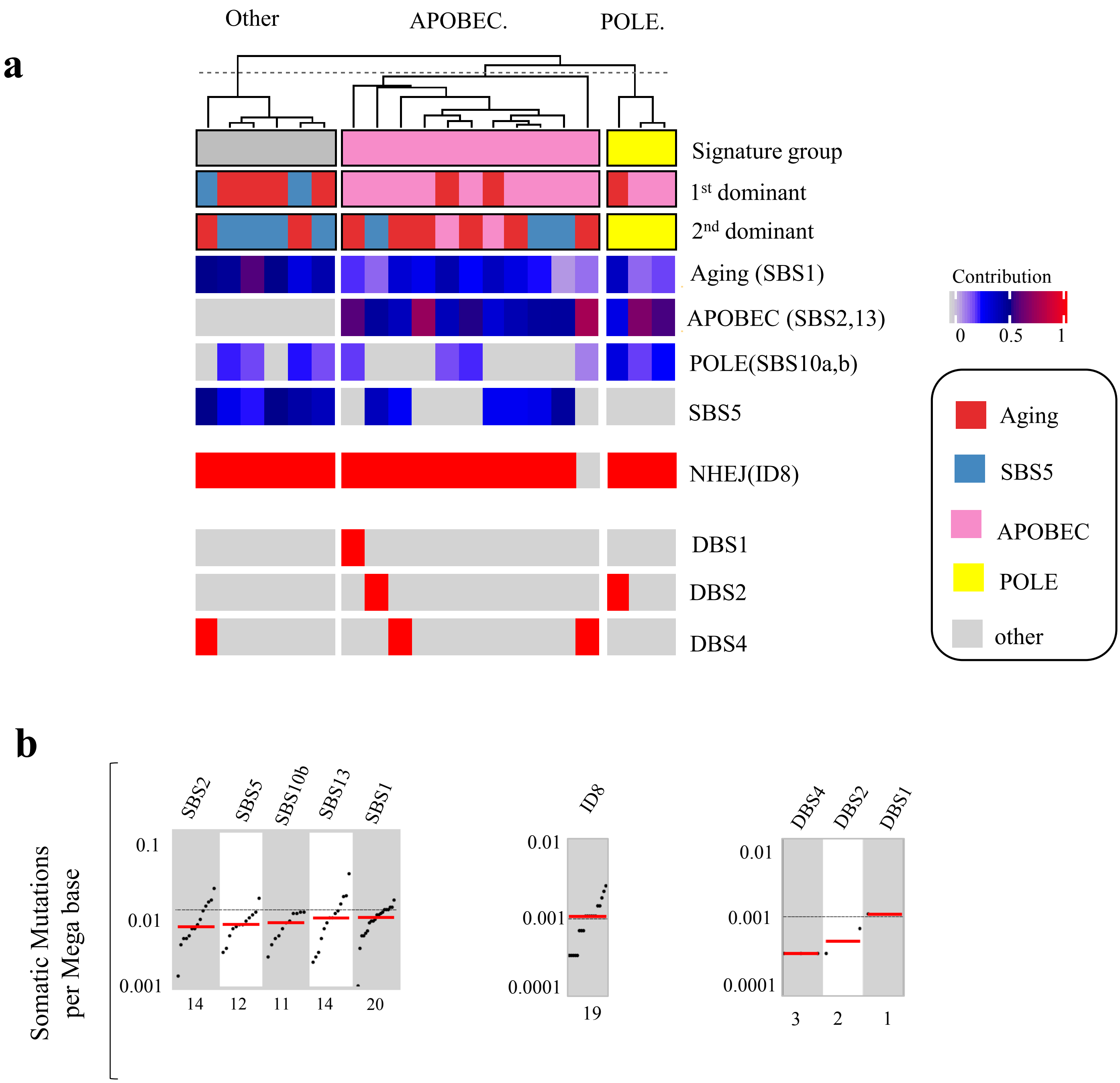

a
Other
APOBEC.
POLE.
Signature group
1st dominant
2nd dominant
Aging (SBS1)
APOBEC (SBS2,13)
POLE(SBS10a,b)
SBS5
NHEJ(ID8)
DBS1
DBS2
DBS4
Contribution
0
0.5
1
Aging
SBS5
APOBEC
POLE
other
b
Somatic Mutations per Mega base
SBS2
SBS5
SBS10b
SBS13
SBS1
0.1
0.01
0.001
14
12
11
14
20
ID8
0.01
0.001
0.0001
19
DBS4
DBS2
DBS1
0.01
0.001
0.0001
3
2
1

Fig. S8

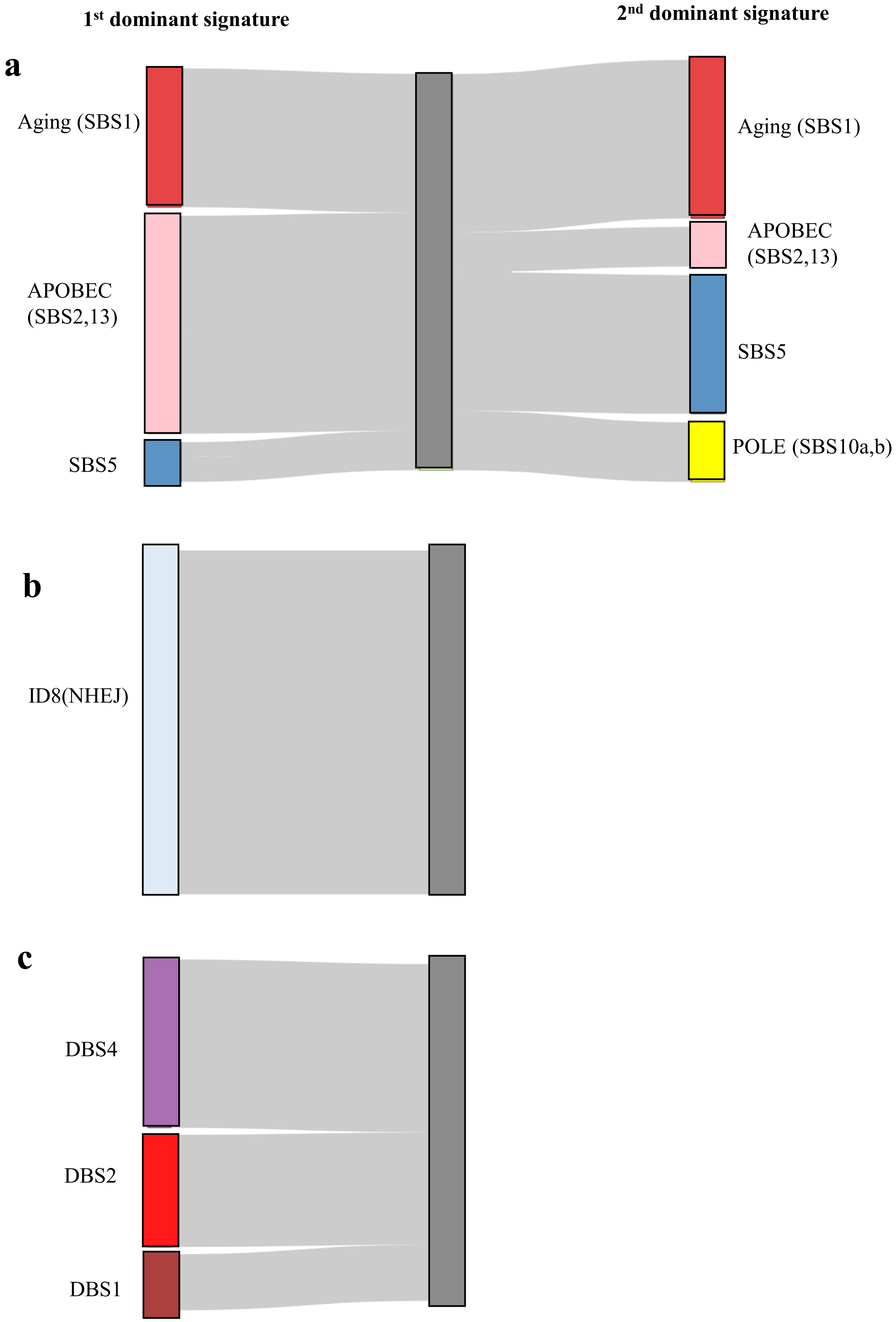
1st dominant signature
2nd dominant signature
a
Aging (SBS1)
APOBEC (SBS2,13)
SBS5
Aging (SBS1)
APOBEC (SBS2,13)
SBS5
POLE (SBS10a,b)
b
ID8(NHEJ)
c
DBS4
DBS2
DBS1

Fig. S9

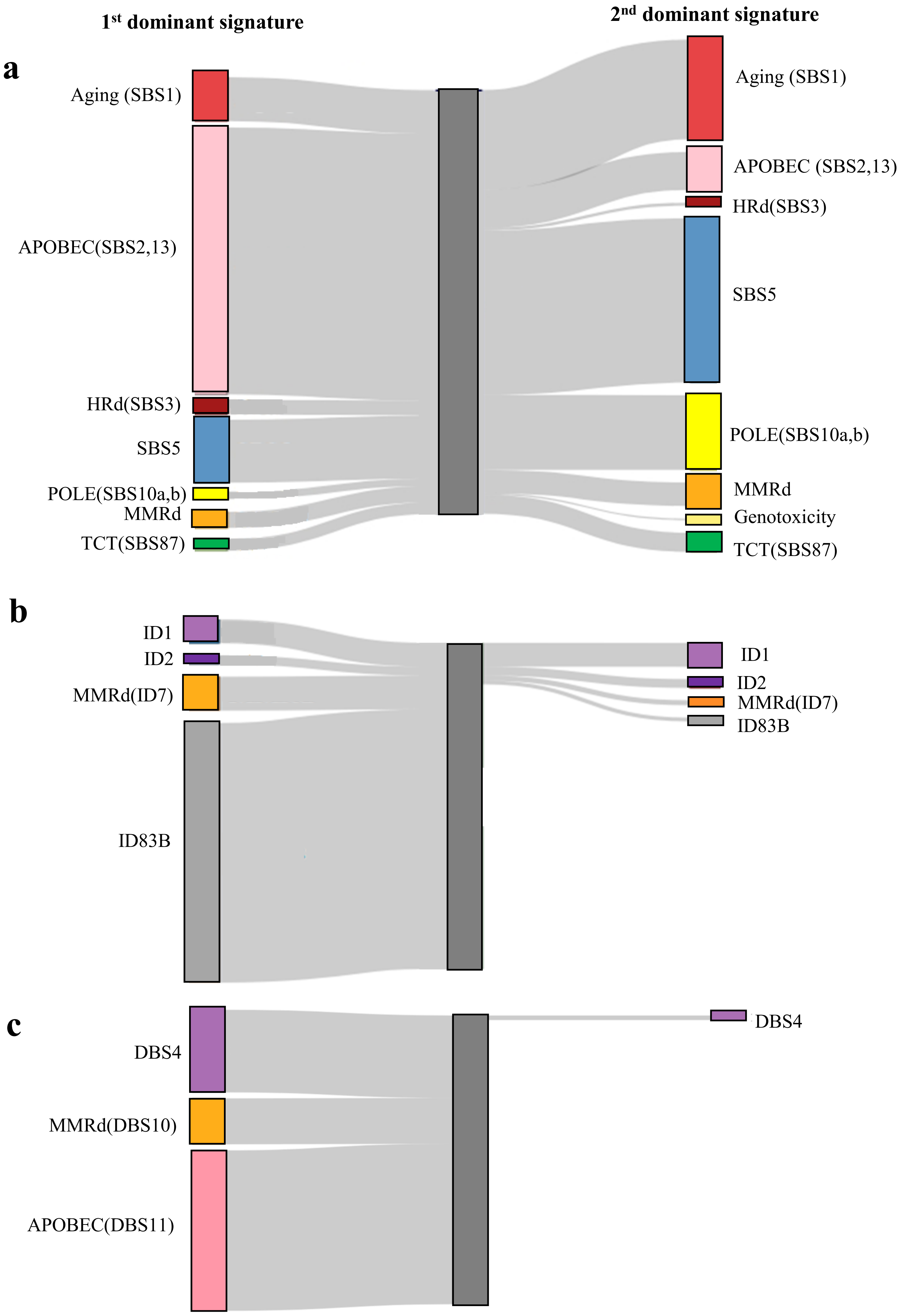
1st dominant signature
2nd dominant signature
a
Aging (SBS1)
APOBEC(SBS2,13)
HRd(SBS3)
SBS5
POLE(SBS10a,b)
MMRd
TCT(SBS87)
Aging (SBS1)
APOBEC (SBS2,13)
HRd(SBS3)
SBS5
POLE(SBS10a,b)
MMRd
Genotoxicity
TCT(SBS87)
b
ID1
ID2
MMRd(ID7)
ID83B
ID1
ID2
MMRd(ID7)
ID83B
c
DBS4
MMRd(DBS10)
APOBEC(DBS11)
DBS4

Fig. S10

a

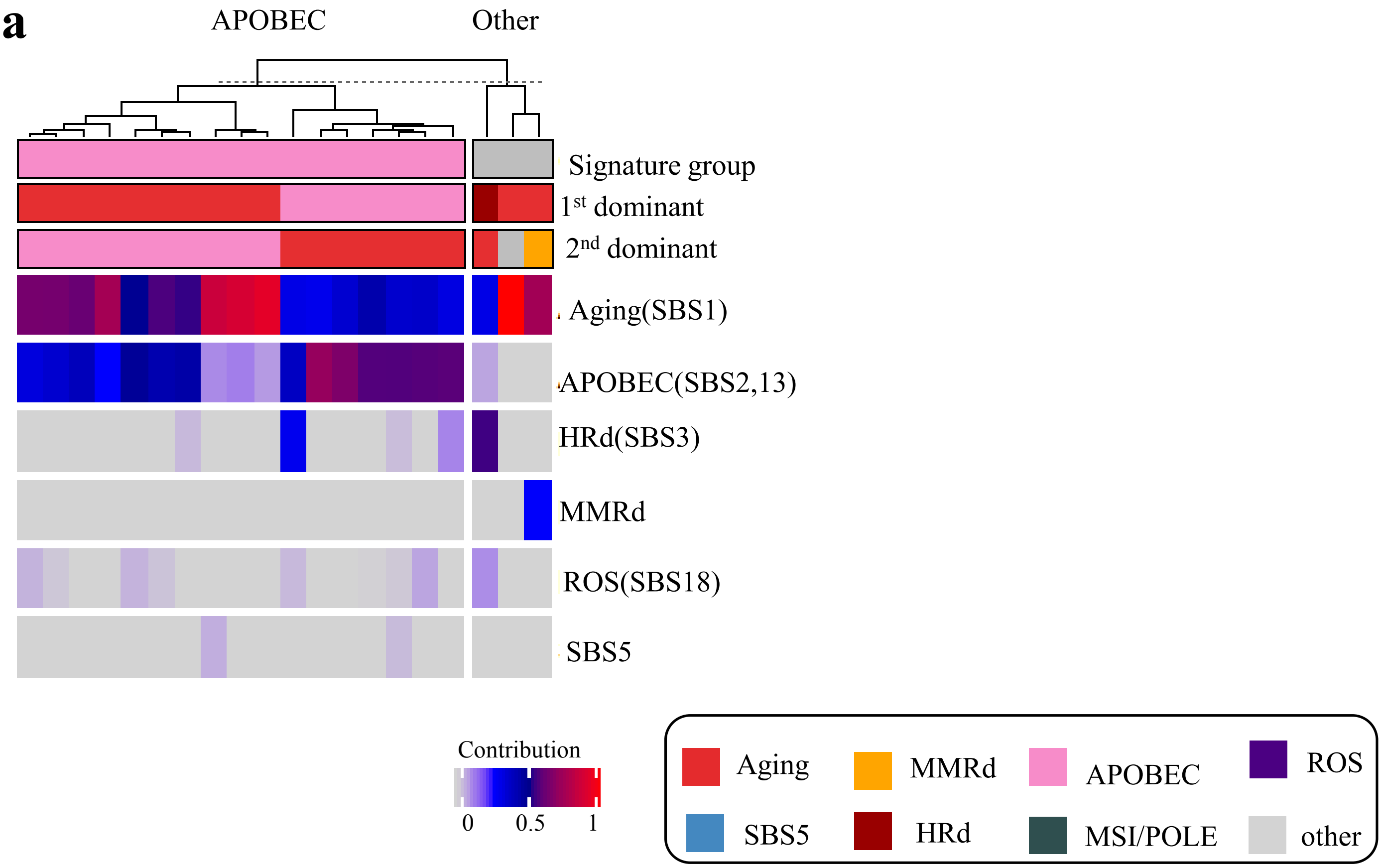
APOBEC
Other
Signature group
1st dominant
2nd dominant
Aging(SBS1)
APOBEC(SBS2,13)
HRd(SBS3)
MMRd
ROS(SBS18)
SBS5
Contribution
0
0.5
1
Aging
MMRd
APOBEC
ROS
SBS5
HRd
MSI/POLE
other

b

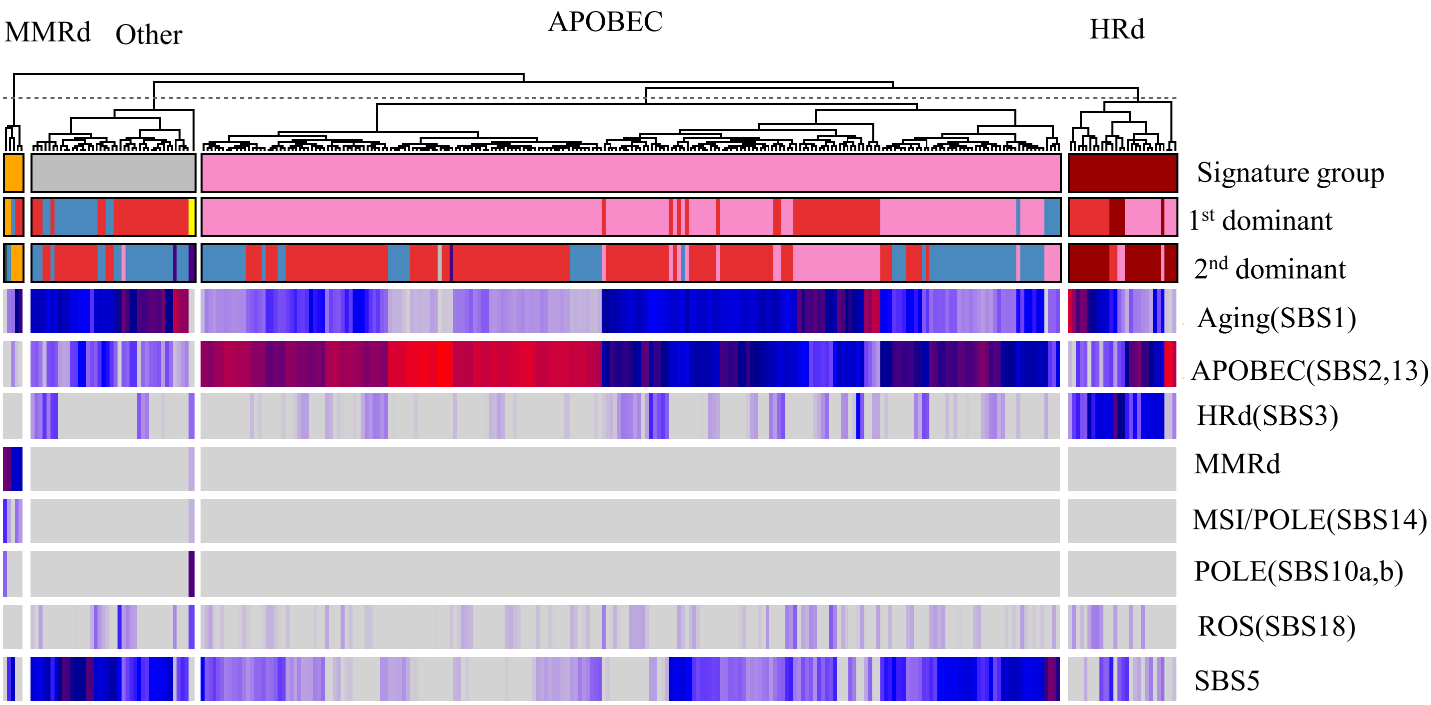
MMRd
Other
APOBEC
HRd
Signature group
1st dominant
2nd dominant
Aging(SBS1)
APOBEC(SBS2,13)
HRd(SBS3)
MMRd
MSI/POLE(SBS14)
POLE(SBS10a,b)
ROS(SBS18)
SBS5

Fig. S11

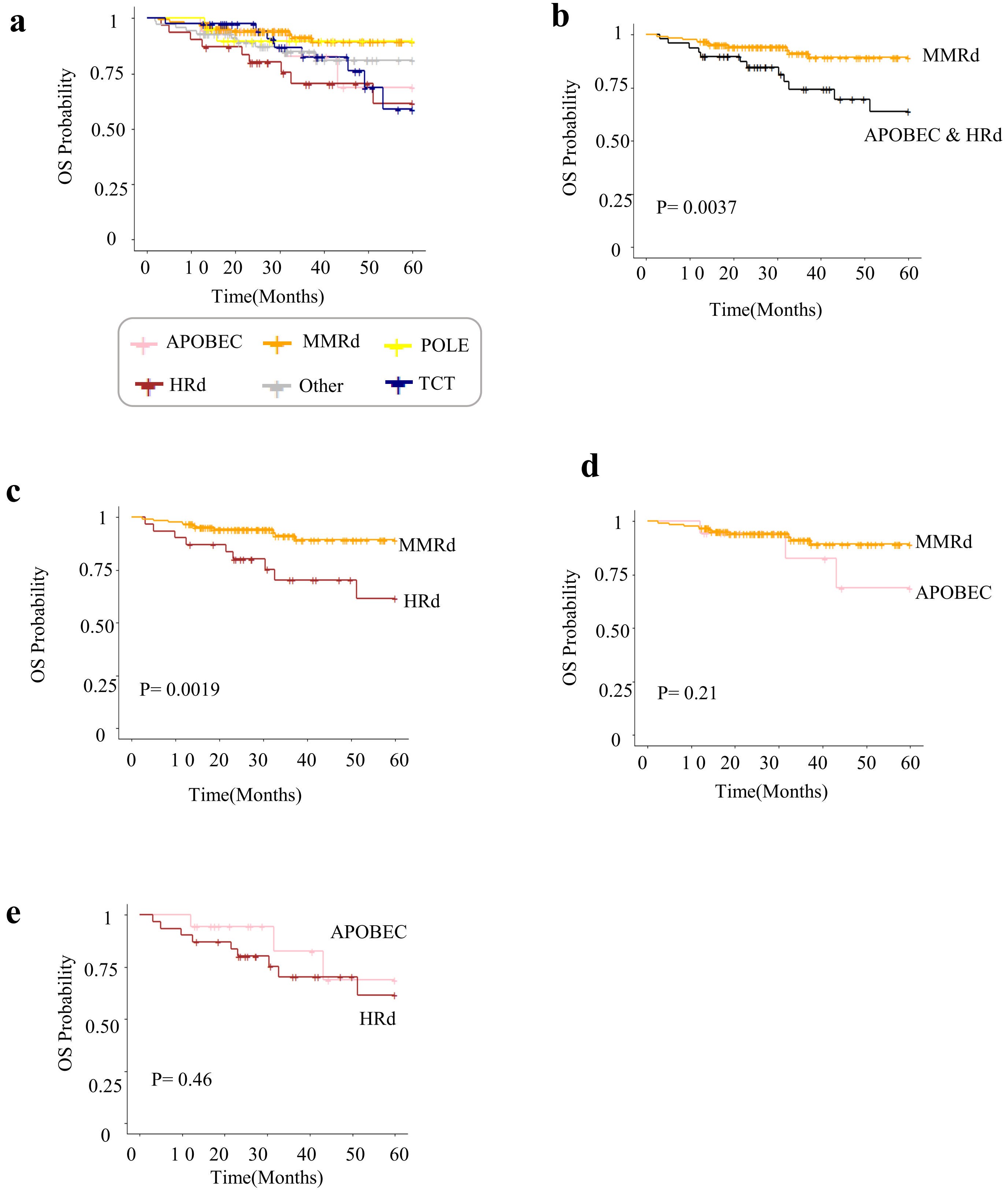
a
OS Probability
1
0.75
0.50
0.25
0
0
1 0
20
30
40
50
60
Time(Months)
APOBEC
MMRd
POLE
HRd
Other
TCT
b
MMRd
APOBEC & HRd
P= 0.0037
c
MMRd
HRd
P= 0.0019
d
MMRd
APOBEC
P= 0.21
e
APOBEC
HRd
P= 0.46

Fig. S12

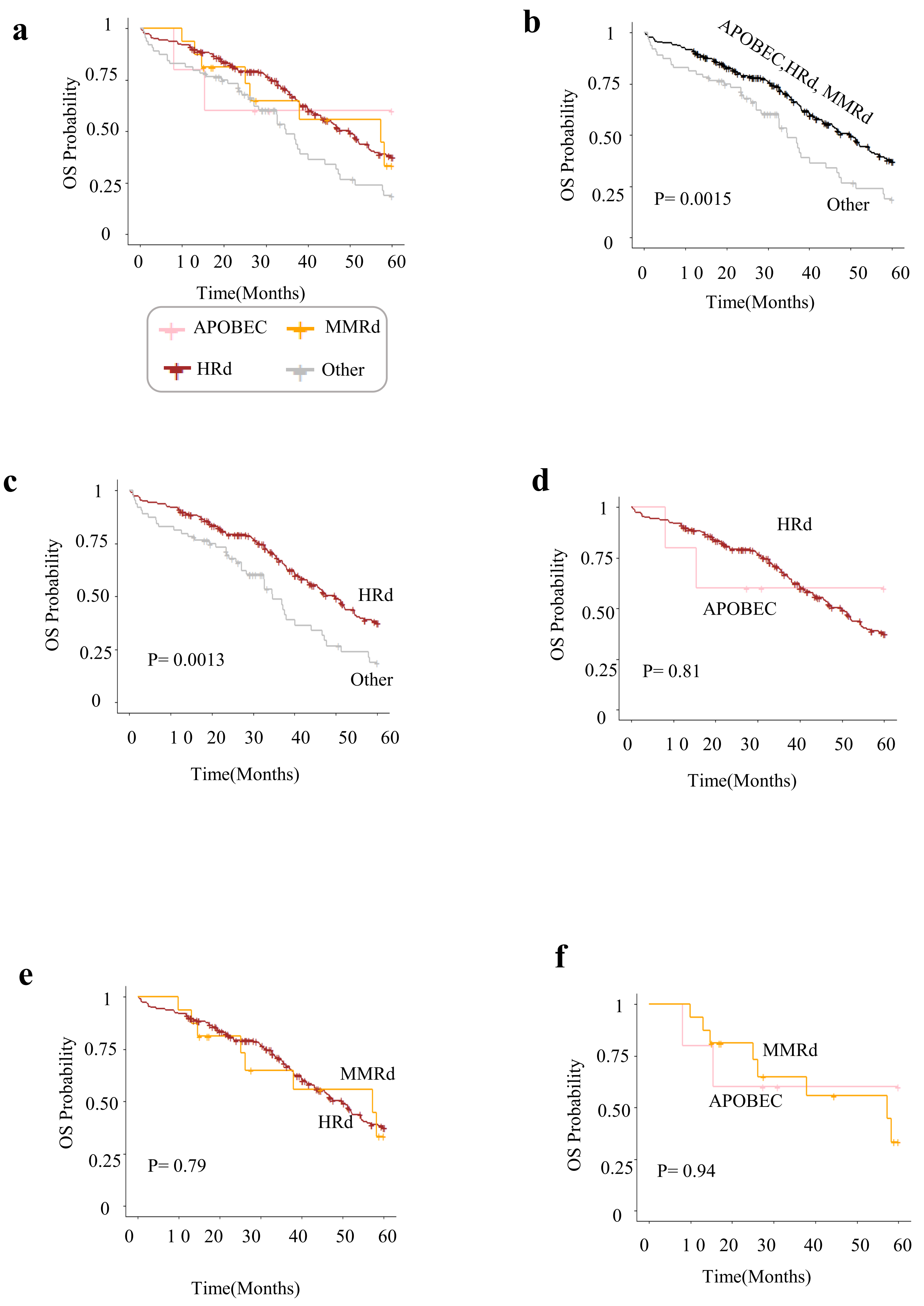

a
OS Probability
1
0.75
0.50
0.25
0
0
1 0
20
30
40
50
60
Time(Months)
APOBEC
MMRd
HRd
Other
b
APOBEC,HRd, MMRd
P= 0.0015
Other
c
HRd
P= 0.0013
Other
d
HRd
APOBEC
P= 0.81
e
MMRd
HRd
P= 0.79
f
MMRd
APOBEC
P= 0.94

Fig. S13

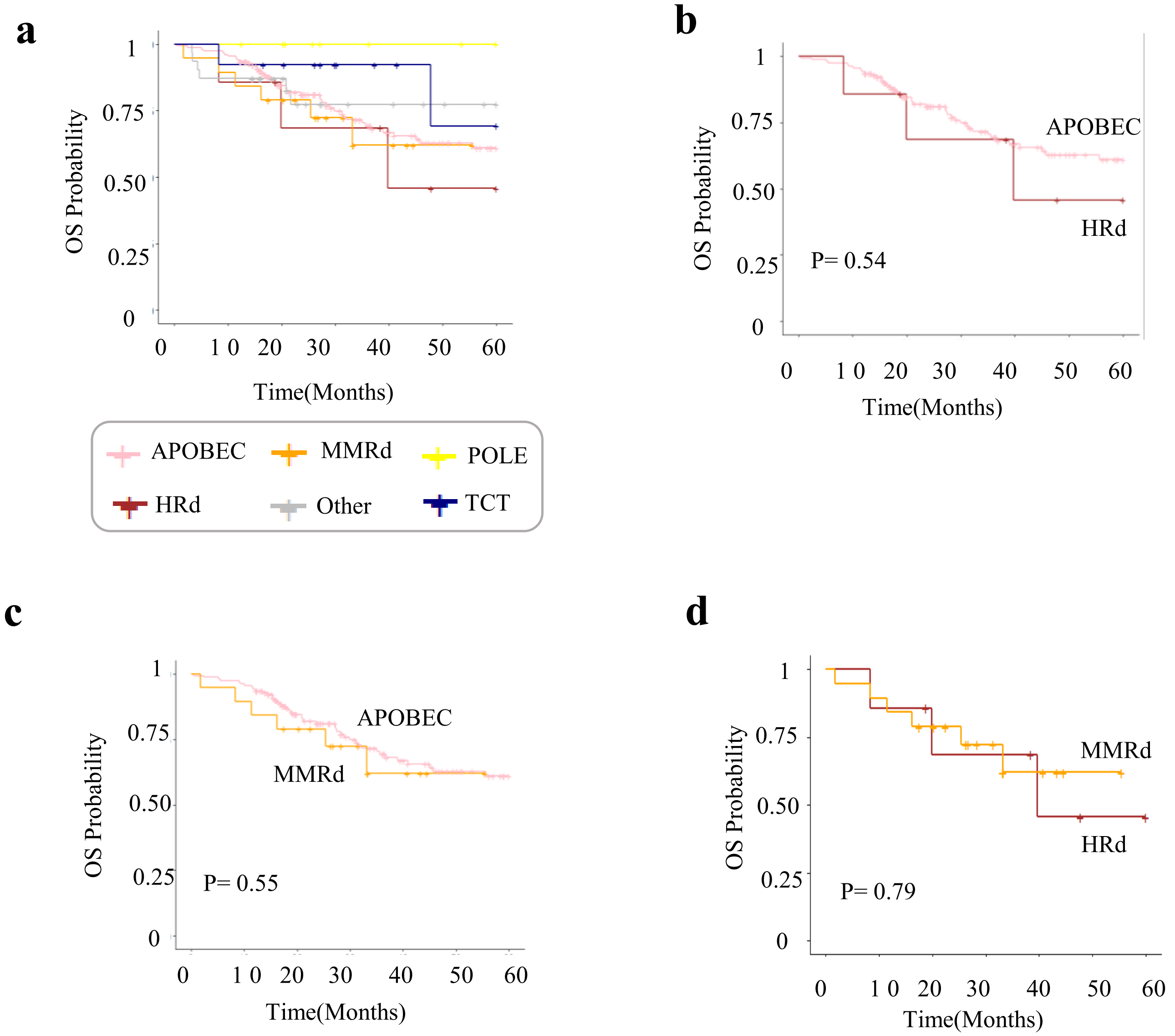
a
1
0.75
0.50
0.25
0
OS Probability
0
1 0
20
30
40
50
60
Time(Months)
APOBEC
MMRd
POLE
HRd
Other
TCT
b
1
0.75
0.50
0.25
0
OS Probability
APOBEC
HRd
P= 0.54
0
1 0
20
30
40
50
60
Time(Months)
c
1
0.75
0.50
0.25
0
OS Probability
APOBEC
MMRd
P= 0.55
0
1 0
20
30
40
50
60
Time(Months)
d
1
0.75
0.50
0.25
0
OS Probability
MMRd
HRd
P= 0.79
0
1 0
20
30
40
50
60
Time(Months)

Fig. S14

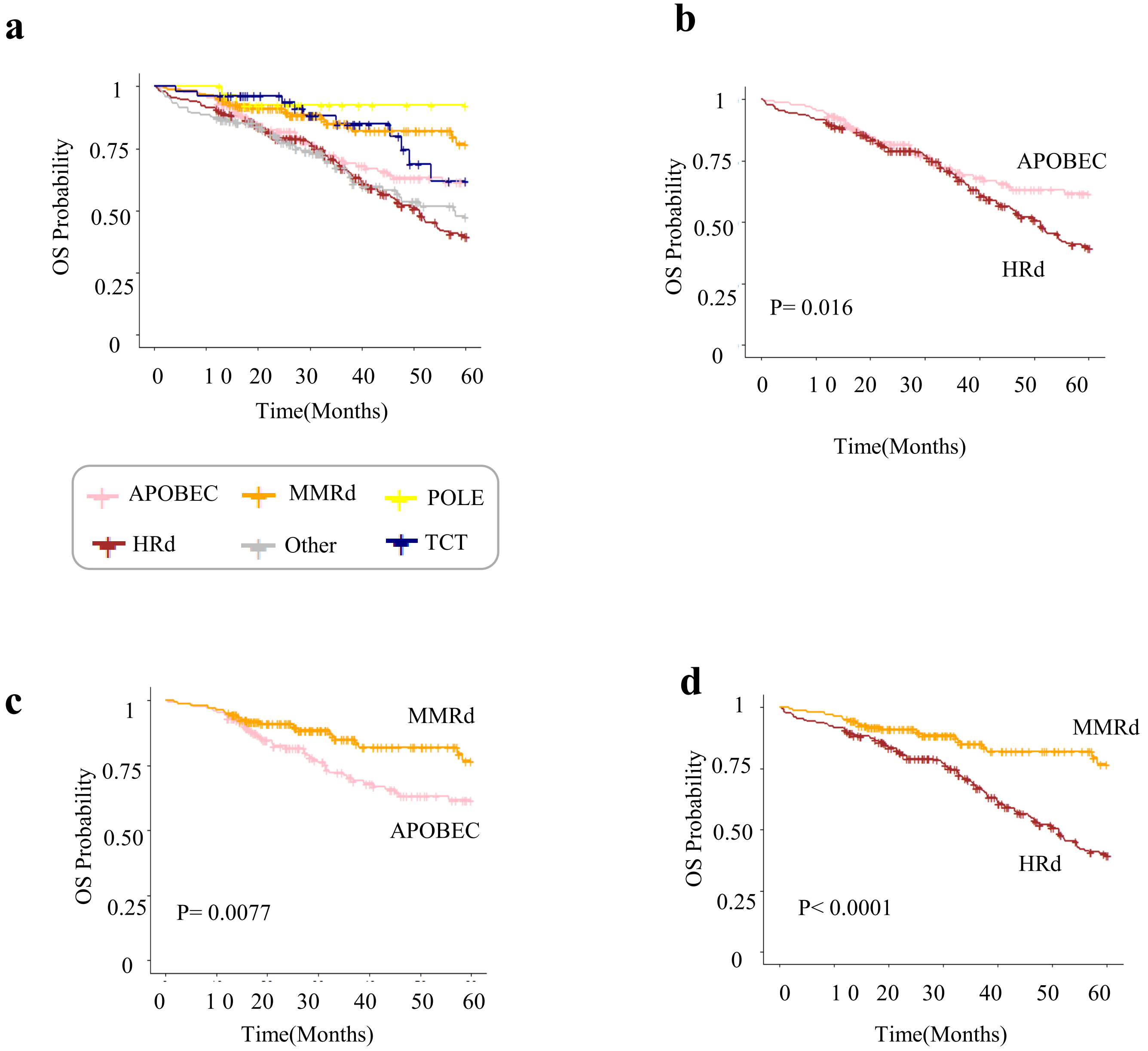
a
OS Probability
1
0.75
0.50
0.25
0
0
1 0
20
30
40
50
60
Time(Months)
APOBEC
MMRd
POLE
HRd
Other
TCT
b
OS Probability
APOBEC
HRd
P= 0.016
Time(Months)
c
OS Probability
MMRd
APOBEC
P= 0.0077
Time(Months)
d
OS Probability
MMRd
HRd
P< 0.0001
Time(Months)

Fig. S15

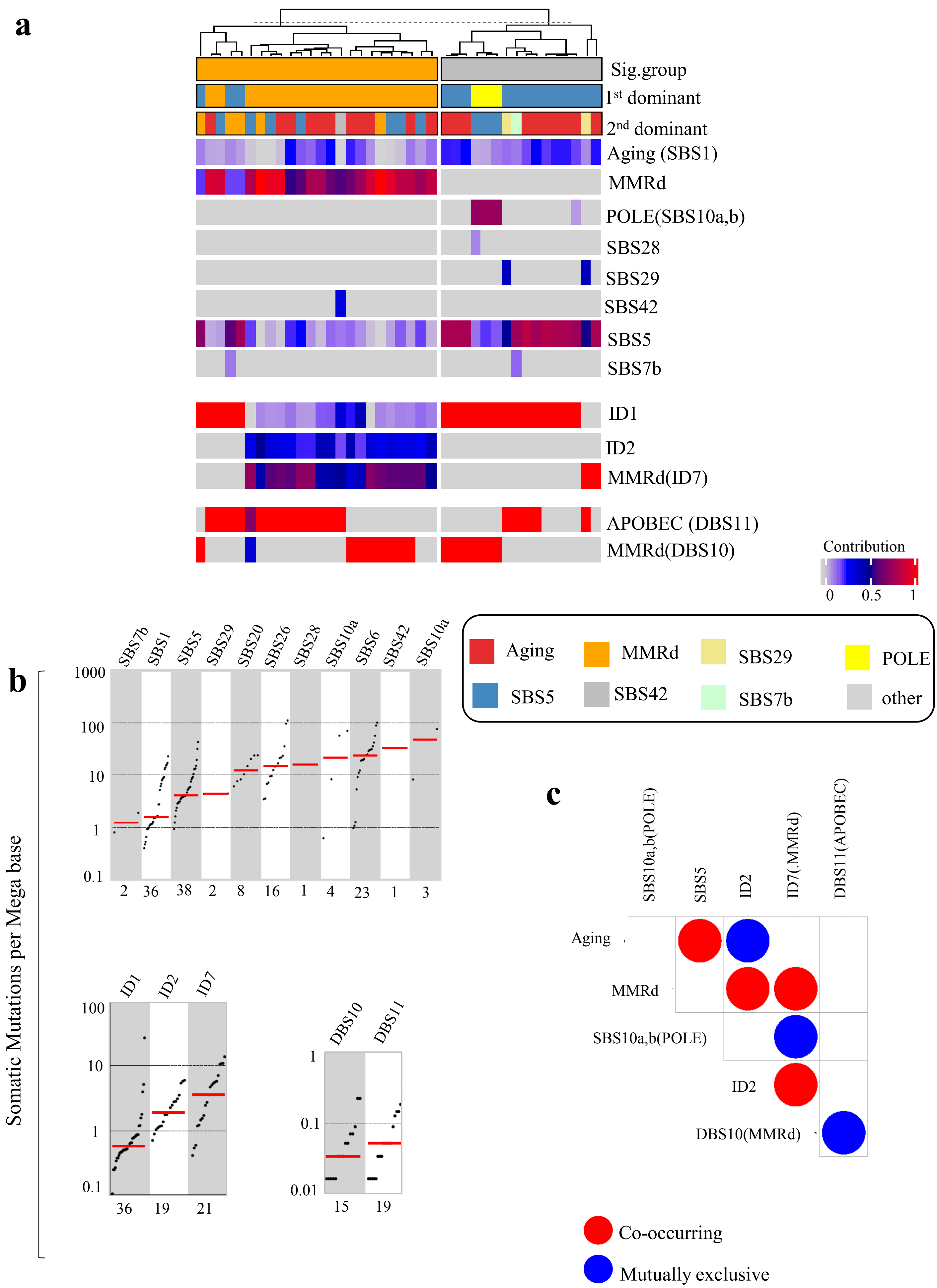
a
MMRd
Other
Sig.group
1st dominant
2nd dominant
Aging (SBS1)
MMRd
POLE(SBS10a,b)
SBS28
SBS29
SBS42
SBS5
SBS7b
ID1
ID2
MMRd(ID7)
APOBEC (DBS11)
MMRd(DBS10)
Contribution
0
0.5
1
Aging
MMRd
SBS29
POLE
SBS5
SBS42
SBS7b
other
b
Somatic Mutations per Mega base
SBS7b
SBS1
SBS5
SBS29
SBS20
SBS26
SBS28
SBS10a
SBS6
SBS42
SBS10a
1000
100
10
1
0.1
2
36
38
2
8
16
1
4
23
1
3
ID1
ID2
ID7
100
10
1
0.1
36
19
21
DBS10
DBS11
1
0.1
0.01
15
19
c
SBS10a,b(POLE)
SBS5
ID2
ID7(.MMRd)
DBS11(APOBEC)
Aging
MMRd
SBS10a,b(POLE)
ID2
DBS10(MMRd)
Co-occurring
Mutually exclusive

Fig. S16

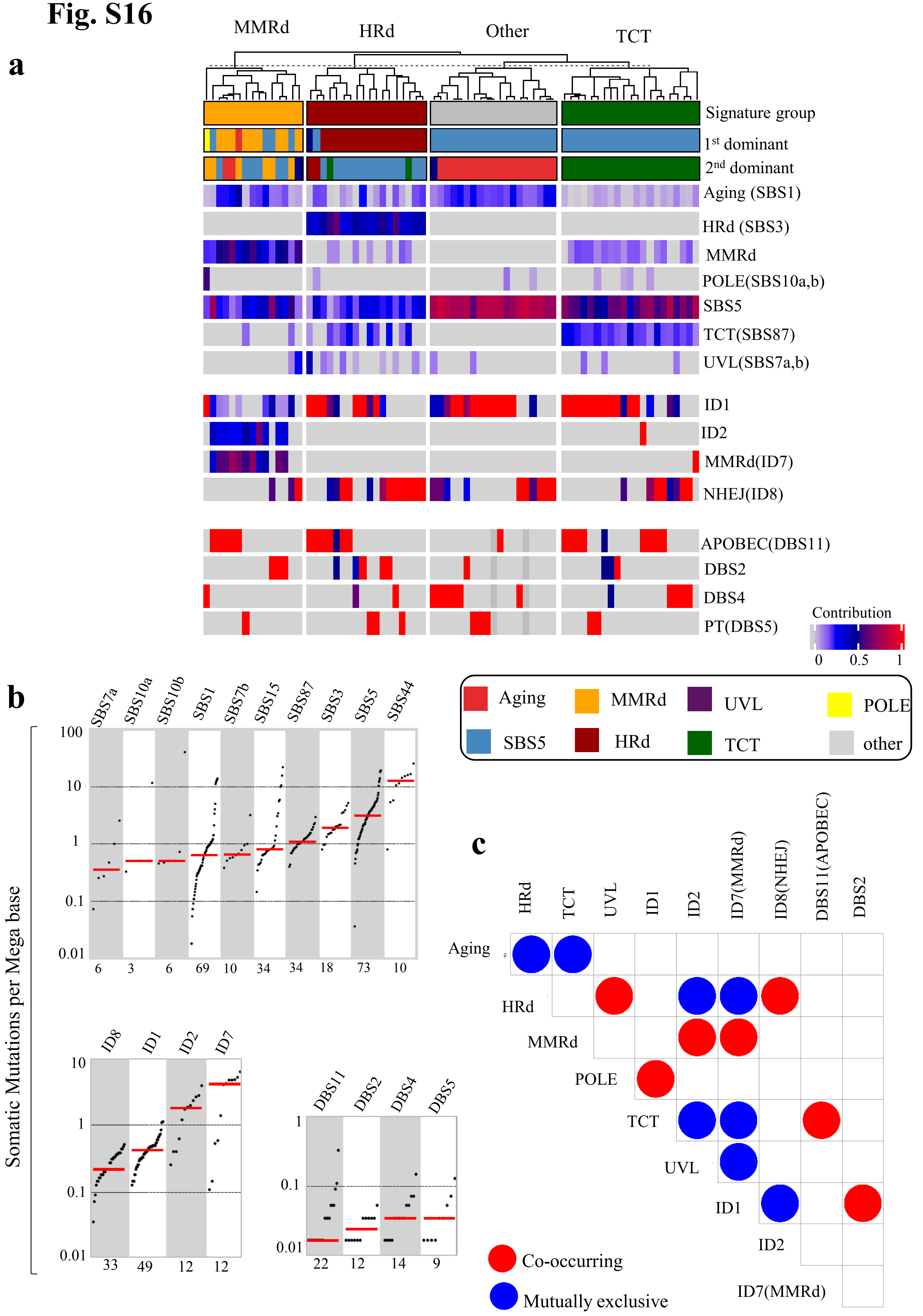
a
MMRd
HRd
Other
TCT
Signature group
1st dominant
2nd dominant
Aging (SBS1)
HRd (SBS3)
MMRd
POLE(SBS10a,b)
SBS5
TCT(SBS87)
UVL(SBS7a,b)
ID1
ID2
MMRd(ID7)
NHEJ(ID8)
APOBEC(DBS11)
DBS2
DBS4
PT(DBS5)
Contribution
0
0.5
1
b
Somatic Mutations per Mega base
SBS7a
SBS10a
SBS10b
SBS1
SBS7b
SBS15
SBS87
SBS3
SBS5
SBS44
100
10
1
0.1
0.01
6
3
6
69
10
34
34
18
73
10
ID8
ID1
ID2
ID7
33
49
12
12
DBS11
DBS2
DBS4
DBS5
22
12
14
9
Aging
MMRd
UVL
POLE
SBS5
HRd
TCT
other
c
HRd
TCT
UVL
ID1
ID2
ID7(MMRd)
ID8(NHEJ)
DBS11(APOBEC)
DBS2
Aging
HRd
MMRd
POLE
TCT
UVL
ID1
ID2
ID7(MMRd)
Co-occurring
Mutually exclusive

Fig. S17

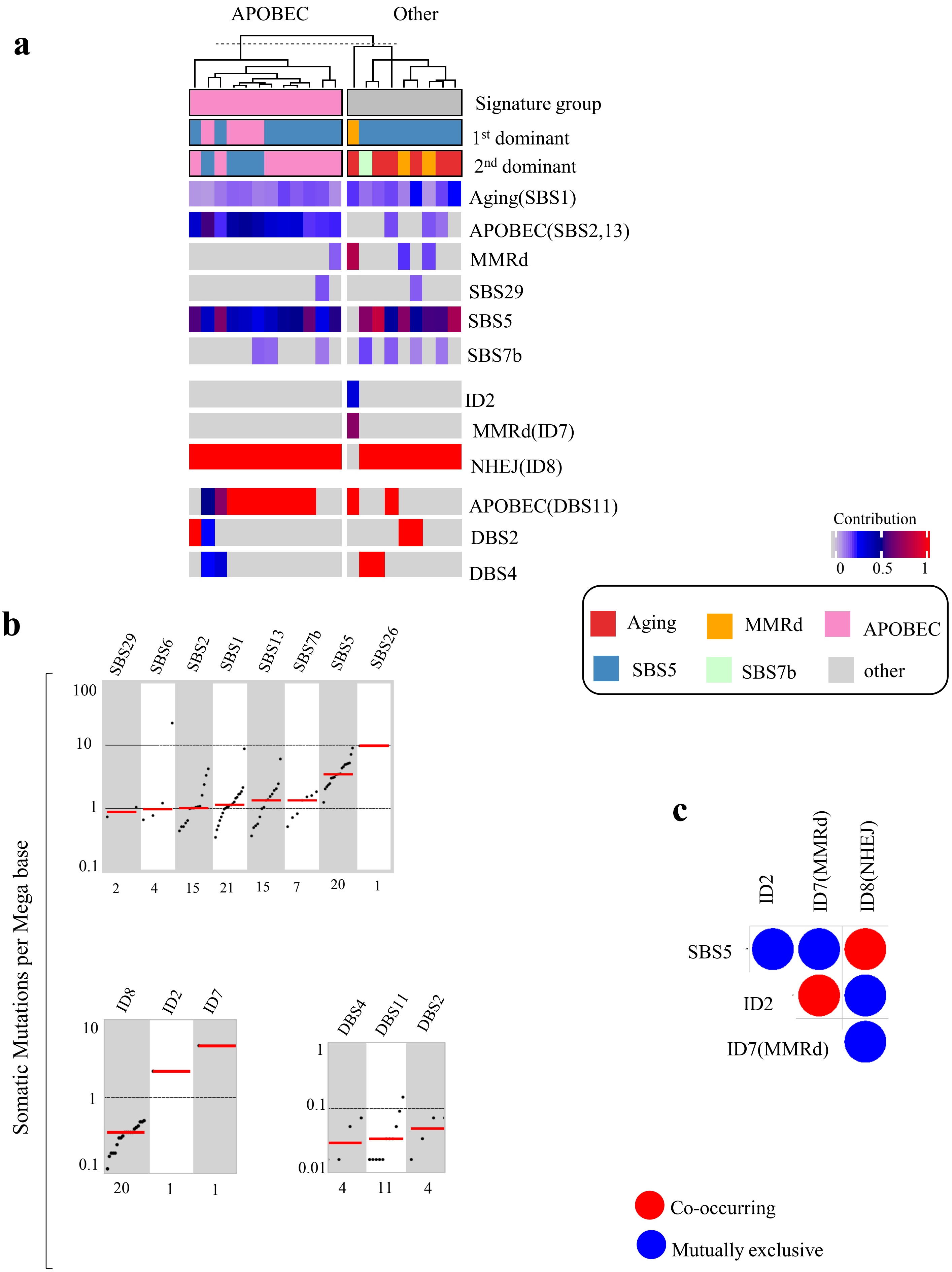
a
APOBEC
Other
Signature group
1st dominant
2nd dominant
Aging(SBS1)
APOBEC(SBS2,13)
MMRd
SBS29
SBS5
SBS7b
ID2
MMRd(ID7)
NHEJ(ID8)
APOBEC(DBS11)
DBS2
DBS4
Contribution
0
0.5
1
Aging
MMRd
APOBEC
SBS5
SBS7b
other
b
Somatic Mutations per Mega base
SBS29
SBS6
SBS2
SBS1
SBS13
SBS7b
SBS5
SBS26
100
10
1
0.1
2
4
15
21
15
7
20
1
ID8
ID2
ID7
10
1
0.1
20
1
1
DBS4
DBS11
DBS2
1
0.1
0.01
4
11
4
c
ID2
ID7(MMRd)
ID8(NHEJ)
SBS5
ID2
ID7(MMRd)
Co-occurring
Mutually exclusive
