# Supplementary material for: Mutational signatures reveal ternary relationships between homologous recombination repair, APOBEC, and mismatch repair in gynecological cancers
Source: J Transl Med. 2022 Feb 2;20:65. doi: 10.1186/s12967-022-03259-0 (PMC8812249; doi:10.1186/s12967-022-03259-0)
Supplement: Supplementary file 3 — Additional file 3. Table S2~S9. P values of mutational signature interactions. [file 12967_2022_3259_MOESM3_ESM.pdf]

Table S2: Interaction of mutational signatures with each other in UCEC WGS tumors analyzed by NMF-based approach. P value of co-occurrence and exclusivity of mutational signatures is measured by hypergeometric test. Also, see Figure1.

Signature_pair1	Signature_pair2	P value_Exclusivity	P value_Coocurence
APOBEC	MMRd	0.02051	0.99531
HRd	MMRd	0.02016	1
SBS8	MMRd	0.00026	1
SBS8	POLE	0.04723	1
APOBEC	ID1	0.03784	0.99326
SBS8	ID1	0.00725	1
MMRd	ID1	0.99942	0.00417
SBS8	ID2	0.00328	1
MMRd	ID2	1	0
ID1	ID2	0.99998	0.00026
SBS8	ID3	0.0156	1
SBS8	MMRd.ID	0.0156	1
MMRd	MMRd.ID	1	0
ID1	MMRd.ID	0.99999	0.00023
ID2	MMRd.ID	1	0
SBS8	NHEJ.ID	3.00E-05	1
NHEJ.ID	AID.APOBEC.DBS	0.03768	0.99552
DBS4	AID.APOBEC.DBS	0.99913	0.00572
DBS9	AID.APOBEC.DBS	0.02254	1
SBS8	DBS2	1	0
MMRd	DBS2	0.00084	1
ID1	DBS2	0.01447	1
ID2	DBS2	0.00735	1
ID3	DBS2	0.02794	1
MMRd.ID	DBS2	0.02794	1
NHEJ.ID	DBS2	0.00011	1
SBS8	DBS4	1	4.00E-05
POLE	DBS4	0.01148	1
MMRd	DBS4	0.00107	0.99989
ID1	DBS4	0.00851	0.99928
ID2	DBS4	0.0031	0.99978
ID3	DBS4	0.02213	0.99776
MMRd.ID	DBS4	0.00224	1
NHEJ.ID	DBS4	0.00304	0.99948
DBS2	DBS4	1	0
SBS8	DBS6	1	0
MMRd	DBS6	0.00255	1
ID1	DBS6	0.02797	1
ID2	DBS6	0.0158	1
ID3	DBS6	0.04869	1
MMRd.ID	DBS6	0.04869	1
NHEJ.ID	DBS6	0.03251	0.99416
DBS2	DBS6	1	4.00E-05
DBS4	DBS6	0.99778	0.01021
APOBEC	DBS9	0.04536	0.98801
ID3	DBS9	0.04058	1

Table S3: Interaction of mutational signatures with each other in UCEC WES tumors analyzed by NMF-based approach. P value of co-occurrence and exclusivity of mutational signatures is measured by hypergeometric test. Also, see Figure2.

Signature_pair1	Signature_pair2	P value_Exclusivity	P value_Coocurence
ID1	ID10	0	1
ID1	ID11	0	1
ID1	ID83B	1	0
ID1	MMRd.DBS	0.99933	0.00127
ID1	Aging	0.99771	0.00832
ID1	APOBEC	0	1
ID1	HRd	0	1
ID1	SBS7b	0.00083	0.99983
ID1	MSI.POLE	1	2.00E-05
ID1	MMRd	1	0
ID1	SBS54	1	2.00E-05
ID1	TCT	0	1
ID10	ID11	0.00267	0.99938
ID10	ID83B	0	1
ID10	SBS7b	0.99781	0.00892
ID10	MMRd	0.01375	0.99333
ID10	SBS54	0.03443	1
ID11	ID83B	0	1
ID11	MMRd.DBS	0.01984	0.98939
ID11	MSI.POLE	0.0168	1
ID11	MMRd	0.0479	0.97042
ID83B	APOBEC	0	1
ID83B	HRd	0	1
ID83B	SBS5	0.98882	0.0331
ID83B	POLE	0	1
ID83B	MMRd	1	0
ID83B	SBS54	1	0
ID83B	TCT	0	1
MMRd.DBS	SBS5	0.00112	0.99968
MMRd.DBS	POLE	0.99853	0.00292
MMRd.DBS	MSI.POLE	1	0
MMRd.DBS	TCT	0.04045	0.9758
Aging	MMRd	0.00021	0.99997
Aging	TCT	0	1
APOBEC	HRd	0.99997	0.00012
APOBEC	MMRd	0	1
APOBEC	SBS54	0.01823	1
HRd	SBS5	0	1
HRd	MMRd	0	1
SBS5	POLE	1.00E-05	1
SBS5	MMRd	0.97844	0.04798
SBS7b	POLE	0.04241	0.99038
POLE	MSI.POLE	0.99946	0.00266
POLE	MMRd	0.00025	0.99989
MSI.POLE	MMRd	0.99999	1.00E-04
MSI.POLE	AzT	0.99875	0.02291
MMRd	SBS54	1	0
SBS54	TCT	0.04271	0.99034

Table S4: Interaction of mutational signatures with each other in UCEC WGS tumors analyzed by multivariate-based approach. P value of co-occurrence and exclusivity of mutational signatures is measured by hypergeometric test. Also, see Additional file1: Fig. S3.

Signature_pair1	Signature_pair2	P value_Exclusivity	P value_Coocurence
Aging	MMRd	0.03321	1
APOBEC	HRd	1	0.00012
APOBEC	SBS5	1	0.00012
APOBEC	MMRd	0	1
APOBEC	SBS8	0.99899	0.01181
HRd	SBS5	0.99994	0.00033
HRd	MMRd	0	1
HRd	SBS8	0.99915	0.00373
HRd	POLE	0.0051	1
HRd	MSI.POLE	0.01531	1
SBS5	MMRd	0.00165	0.99976
SBS5	SBS8	1	0
SBS5	POLE	0.0051	1
MMRd	POLE	0.99987	0.00392
MMRd	MSI.POLE	1	0.00089
MMRd	ROS	0.00462	0.99932

Table S5: Interaction of mutational signatures with each other in ovarian WGS tumors analyzed by NMF-based approach. P value of co-occurrence and exclusivity of mutational signatures is measured by hypergeometric test. Also, see Figure3.

Signature_pair1	Signature_pair2	P value_Exclusivity	P value_Coocurence
APOBEC	HRd	0.9956	0.01133
APOBEC	SBS8	0.99929	0.00224
APOBEC	SBS39	0.01644	0.9983
APOBEC	SBS60	1	0.00467
APOBEC	DBS2	1	0
APOBEC	DBS4	0.99588	0.01132
APOBEC	DBS6	0.99925	0.00228
HRd	SBS39	2.00E-05	1
HRd	SBS60	1	0.03661
HRd	ID1	0	1
HRd	ID2	0.00044	1
HRd	NHEJ.ID	0.99969	0.00186
HRd	ID83C.ID	9.00E-05	0.99998
HRd	DBS2	0.99994	0.00023
HRd	DBS4	0.99992	3.00E-04
HRd	DBS6	0.99835	0.00453
SBS5	SBS8	1	0.0058
SBS5	DBS2	0.99926	0.01165
SBS5	DBS6	1	0.0353
SBS8	SBS39	0.99704	0.01845
SBS8	ID1	0.99726	0.00883
SBS8	NHEJ.ID	0.99021	0.03227
SBS8	ID83C.ID	0.9994	0.0018
SBS8	DBS2	1	0
SBS8	DBS4	1	0
SBS8	DBS6	0.99987	0.00044
SBS8	DBS9	0.99136	0.02219
Aflatoxin	DBS2	0.02912	1
MMRd	Genotoxicity	1	0.00637
BERd	DBS2	0.01185	0.99873
PT	PT.DBS	1	1.00E-05
SBS60	DBS6	1	0.01199
ID1	ID2	1	1.00E-05
ID1	ID83C.ID	1	0
ID2	ID83C.ID	0.99405	0.03589
NHEJ.ID	ID83C.ID	0.00186	0.99969
NHEJ.ID	DBS2	0.99571	0.01614
NHEJ.ID	DBS4	0.99997	2.00E-04
NHEJ.ID	DBS6	0.99987	0.00118
ID83C.ID	DBS2	0.99173	0.02038
DBS2	DBS4	1	0
DBS2	PT.DBS	0.99549	0.01784
DBS2	DBS6	1	0
DBS2	DBS9	0.99314	0.02012
DBS4	DBS6	0.99996	0.00017
DBS4	DBS9	0.9801	0.04739
DBS6	DBS9	0.98439	0.03625

Table S6: Interaction of mutational signatures with each other in ovarian WES tumors analyzed by NMF-based approach. P value of co-occurrence and exclusivity of mutational signatures is measured by hypergeometric test. Also, see Figure4.

Signature_pair1	Signature_pair2	P value_Exclusivity	P value_Coocurence
Aging	HRd	0.99953	0.00511
Aging	SBS49	0.0386	0.99967
Aging	SBS53	0.0386	0.99967
APOBEC	SBS5	0.00044	0.99986
APOBEC	AID.APOBEC.DBS	0.99936	0.00188
HRd	SBS5	0	1
HRd	SBS7b	0.01979	0.9927
HRd	MMRd	0.00767	0.99665
HRd	DBS2	1	1.00E-05
HRd	DBS4	0.99749	0.0097
HRd	DBS9	0.99958	0.00122
SBS5	DBS2	0.04646	0.9814
SBS5	DBS4	0.01837	0.99324
SBS7b	ID83B	0.99876	0.00437
SBS7b	AID.APOBEC.DBS	0.99978	0.00098
MMRd	DBS6	0.01614	0.99803
SBS49	SBS53	0.99999	0.00972
ID14	ID83B	0.01309	0.99616
ID16	ID83B	0	1
ID83B	AID.APOBEC.DBS	0.99998	7.00E-05
DBS2	DBS6	0.04239	1
DBS2	DBS9	0.00014	1
DBS4	DBS9	0.00404	0.99936
DBS6	DBS9	0.01159	0.99789
DBS6	AID.APOBEC.DBS	0.00297	1
DBS9	AID.APOBEC.DBS	2.00E-05	1

Table S7: Interaction of mutational signatures with each other in ovarian WGS tumors analyzed by multivariate-based approach. P value of co-occurrence and exclusivity of mutational signatures is measured by hypergeometric test. Also, see Additional file 1: Fig. S6.

Signature_pair1	Signature_pair2	P value_Exclusivity	P value_Coocurence
Aging	MMRd	0.01274	1
APOBEC	SBS8	0.99995	0.00115
HRd	SBS5	0.99884	0.01184
HRd	MMRd	0.00229	1
HRd	SBS8	0.9997	0.00371
HRd	ROS	0.00804	0.9992
SBS5	SBS8	1	0
SBS5	ROS	3.00E-04	0.99996
SBS8	ROS	0	1

Table S8: Interaction of mutational signatures with each other in cervical WES tumors analyzed by NMF-based approach. P value of co-occurrence and exclusivity of mutational signatures is measured by hypergeometric test. Also, see Figure5.

Signature_pair1	Signature_pair2	P value_Exclusivity	P value_Coocurence
Aging	APOBEC	1	0.0217
Aging	MMRd	0.03439	1
Aging	TCT	0.00908	1
APOBEC	SBS5	0.04996	1
APOBEC	POLE	0.99647	0.00937
APOBEC	MMRd	0.00467	0.99854
APOBEC	TCT	0.00326	0.99926
APOBEC	ID1	0.04659	0.98164
APOBEC	AID.APOBEC.DBS	0.99807	0.01086
HRd	SBS5	0.03143	0.99614
HRd	DBS4	1	1.00E-05
POLE	TCT	0	1
POLE	ID2	0.02358	0.99496
POLE	MMRd.ID	0.04974	0.97929
MMRd	TCT	0.99964	0.00159
MMRd	ID2	0.9997	0.00203
MMRd	MMRd.ID	0.98523	0.04078
Genotoxicity	MMRd.DBS	1	0.04498
ID1	ID2	0.99947	0.00344
ID1	ID83B	0	1
ID2	MMRd.ID	1	3.00E-05
ID2	ID83B	0	1
ID2	MMRd.DBS	0.99742	0.02372
MMRd.ID	ID83B	0	1
ID83B	AID.APOBEC.DBS	0.98432	0.03867

Table S9: Interaction of mutational signatures with each other in (**A**) uterine, (**B**) ovarian and (**C**) cervical cell lines analyzed by NMF-based approach. P value of co-occurrence and exclusivity of mutational signatures is measured by hypergeometric test. Also, see Additional file 1: Fig. S15, S16,S17.

A

Signature_pair1	Signature_pair2	P value_Exclusivity	P value_Coocurence
Aging	SBS5	1	0.00769
Aging	ID2	0.04241	1
MMRd	POLE	0.01991	1
MMRd	ID2	1	0
MMRd	MMRd.ID	1	4.00E-05
POLE	MMRd.ID	0.04241	1
ID2	MMRd.ID	1	0
MMRd.DBS	AID.APOBEC.DBS	6.00E-05	1

B

Signature_pair1	Signature_pair2	P value_Exclusivity	P value_Coocurence
Aging	HRd	0.04405	0.99719
Aging	TCT	0.04261	1
HRd	UVL	0.99892	0.00719
HRd	ID2	0.02386	1
HRd	MMRd.ID	0.02386	1
HRd	NHEJ.ID	0.99158	0.03319
UVL	ID2	0.04851	1
UVL	MMRd.ID	0.04851	1
POLE	ID1	1	0.03355
MMRd	TCT	0.9999	0.00061
MMRd	ID2	1	0.00015
MMRd	MMRd.ID	0.99985	0.00244
TCT	ID2	0.02271	0.99669
TCT	MMRd.ID	0.02271	0.99669
TCT	AID.APOBEC.DBS	0.98546	0.04794
ID1	NHEJ.ID	0.00039	0.99995
ID1	DBS2	0.99498	0.04305
ID2	MMRd.ID	1	0
ID2	NHEJ.ID	3.00E-04	1
MMRd.ID	NHEJ.ID	3.00E-04	1
DBS4	AID.APOBEC.DBS	0.0035	1
PT.DBS	AID.APOBEC.DBS	0.03134	1

C

Signature_pair1	Signature_pair2	P value_Exclusivity	P value_Coocurence
SBS5	ID2	0.04762	1
SBS5	MMRd	0.04762	1
SBS5	NHEJ	1	0.04762
ID2	MMRd	1	0.04762
ID2	NHEJ	0.04762	1
MMRd	NHEJ	0.04762	1
